# *REC8* is a novel tumor suppressor gene epigenetically robustly targeted by the PI3K pathway in thyroid cancer

**DOI:** 10.18632/oncotarget.5391

**Published:** 2015-10-13

**Authors:** Dingxie Liu, Xiaopei Shen, Guangwu Zhu, Mingzhao Xing

**Affiliations:** ^1^ Laboratory for Cellular and Molecular Thyroid Research, Division of Endocrinology, Diabetes & Metabolism, Department of Medicine, Johns Hopkins University School of Medicine, Baltimore, MD 21287, USA

**Keywords:** REC8, thyroid cancer, tumor suppressor gene, methylation, thyroid tumorigenesis

## Abstract

The role of the PI3K pathway in human cancer has been well established, but much of its molecular mechanism, particularly the epigenetic aspect, remains to be defined. We hypothesized that aberrant methylation and hence altered expression of certain unknown important genes induced by the genetically activated PI3K pathway signaling is a major epigenetic mechanism in human tumorigenesis. Through a genome-wide search for such genes that were epigenetically controlled by the PI3K pathway in thyroid cancer cells, we found a wide range of genes with broad functions epigenetically targeted by the PI3K pathway. The most prominent among these genes was *REC8*, classically known as a meiotic-specific gene, which we found to be robustly down-regulated by the PI3K pathway through hypermethylation. *REC8* hypermethylation was strongly associated with genetic alterations and activities of the PI3K pathway in thyroid cancer cell lines, thyroid cancer tumors, and some other human cancers; it was also associated with poor clinicopathological outcomes of thyroid cancer, including advanced disease stages and patient mortality. Demethylating the hypermethylated *REC8* gene restored its expression in thyroid cancer cells in which the PI3K pathway was genetically over-activated and induced expression of REC8 protein inhibited the proliferation and colony formation of these cells. These findings are consistent with *REC8* being a novel major *bona fide* tumor suppressor gene and a robust epigenetic target of the PI3K pathway. Aberrant inactivation of *REC8* through hypermethylation by the PI3K pathway may represent an important mechanism mediating the oncogenic functions of the PI3K pathway.

## INTRODUCTION

Follicular cell-derived thyroid cancer is a common endocrine malignancy, which histologically consists of papillary thyroid cancer (PTC), follicular thyroid cancer (FTC) and anaplastic thyroid cancer (ATC) [1–3]. Among the different types, PTC and FTC are differentiated thyroid cancer (DTC) with a generally good prognosis, whereas ATC is undifferentiated with a generally dismal prognosis [4]. Like the MAP kinase pathway aberrantly activated by the *BRAF* mutation that plays a fundamental role in thyroid tumorigenesis, particularly in PTC [5], aberrantly activated RAS/PI3K/AKT pathway (PI3K pathway) is another fundamental mechanism in thyroid tumorigenesis, particularly in FTC and ATC [6, 7]. Mutations in the *RAS*, *PIK3CA*, and *PTEN* genes in the PI3K pathway are the main genetic driving force of this pathway in human cancers, including thyroid cancers, particularly FTC and ATC [8, 9]. Nevertheless, beyond the aberrant signaling of the PI3K pathway itself, much is unknown about the specific genes targeted by this pathway in thyroid tumorigenesis, particularly in the epigenetic respect.

DNA methylation is an epigenetic process in which a methyl group is covalently added to the fifth carbon of the cytosine residue and its aberrant occurrence in the promoter areas of genes is a fundamental mechanism of human tumorigenesis [10, 11], including thyroid tumorigenesis [12]. Genes can be hyper- and hypo-methylated, which are usually associated with gene silencing and overexpression, respectively, with serious biological consequences. For example, in thyroid cancer, tumor-suppressor genes, such as *RASSF1A* [13], *TIMP-3* [14] and *RASAL1* [15], are often hypermethylated, whereas oncogenes, such as *HMGB2* and *FGD1* [16] and *MAP17* [17], are often hypomethylated, resulting in their aberrant expression and consequent alterations in important molecular and cellular activities. Using a methylated CpG island amplification/CpG island microarray approach, we previously demonstrated the coupling of the MAP kinase pathway to aberrant methylation of a wide range of genes as a fundamental mechanism in the *BRAF* V600E-promoted tumorigenesis of PTC [16].

In the present study, by performing a genome-wide gene methylation analysis we tested our hypothesis that, as a major mechanism of human tumorigenesis, the PI3K pathway targets epigenetically genes with important oncogenic functions. As a prominent example of such genes, we identified and characterized the *REC8* gene as a novel tumor suppressor gene robustly targeted through aberrant methylation by the PI3K pathway in thyroid cancer and some other cancers, revealing an important novel mechanism mediating the oncogenic function of the PI3K pathway in human tumorigenesis.

## RESULTS

### Genome-wide screening for genes epigenetically targeted by the PI3K pathway in thyroid cancer cells

**Figure 1:**
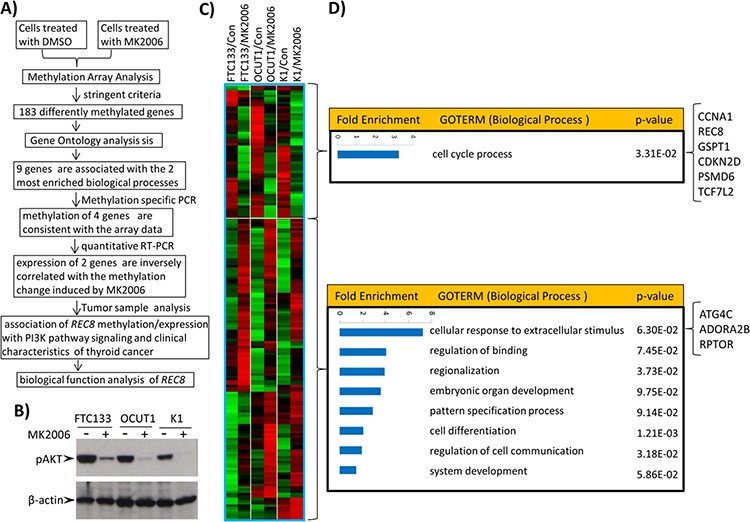
Genome-wide screening of candidate genes regulated by the PI3K/AKT pathway through aberrant gene methylation in thyroid cancer cells and functional annotation of these genes **A.** The overall experimental approach in this study for identification of genes regulated by the PI3K/AKT pathway through aberrant gene methylation in thyroid cancer cells. **B.** Effects of the AKT inhibitor MK2006 on the phosphorylation of AKT in 3 thyroid cancer cell lines. Cells were treated with 1 μM AKT inhibitor MK2006 for 72 h with the inhibitor being replenished every 24 h. **C.** Heatmap of the hypomethylated or hypermethylated genes. The Criteria used to select hypomethylated (upper box) or hypermethylated (lower box) genes after treatment with MK2006 was described in Materials and Methods. A total of 53 probes (57 genes) and 125 probes (126 genes) showed decreased and increased methylation after treatment, respectively. **D.** Gene Ontology analysis of the differentially methylated genes by DAVID functional annotation tool using the default parameters. The biological processes were ranked according to their enrichment folds. The 9 differentially methylated genes that are involved in the biological processes ‘cell cycle’ or ‘cellular response to extracellular stimulus’ are indicated on the right side of the figure.

Figure 1A outlines the overall experimental strategy employed in this study. To identify genes controlled by the PI3K pathway through altering DNA methylation, we used the AKT inhibitor MK2006 to suppress the PI3K pathway in three thyroid cancer cell lines that harbored mutations in the PI3K pathway, including the FTC133 cell harboring *PTEN* deletion, OCUT1 cell harboring *PIK3CA* H1047R^+/+^, and K1 cell harboring *PIK3CA* E542K^+/+^ [18]. We subsequently analyzed genome-wide changes in DNA methylation in the three cell lines using the Illumina Methylation 450K array. MK2006 effectively inhibited the signaling of the PI3K pathway as indicated by the suppression of AKT phosphorylation (Figure 1B), resulting in an increase or decrease in the methylation in 4,793 probe sites in the three cell lines (*p* < 0.01). To ensure both high statistical significance and biological relevance, we selected for analysis only the probes located in transcriptional regulatory regions (including 0–1,500 bp upstream of transcription start site, first exon and enhancer regions) of genes in the present study, with the differentially methylated CpG sites having a *p*-value < 0.005 (univariate paired *T*-test) and fold-change (based on M-value) of > 1.5 in the same direction in all the three cell lines. We identified 57 genes (53 probes) and 126 genes (125 probes) that met these criteria and were significantly hypomethylated and hypermethylated, respectively, after the treatment with MK2006 (Figure 1C and Supplementary Table S1). Gene Ontology analysis using the Entrez IDs of the significantly hypomethylated and hypermethylated genes as inputs, respectively, identified one significantly enriched biological process ontology ‘cell cycle process’ for the hypomethylated genes and 8 biological process ontologies for the hypermethylated genes, including ‘cellular response to extracellular stimulus process’ that had the highest enrichment scores among the 8 ontologies (Figure 1D).

### Validation of the methylation microarray results by quantitative PCR

**Figure 2:**
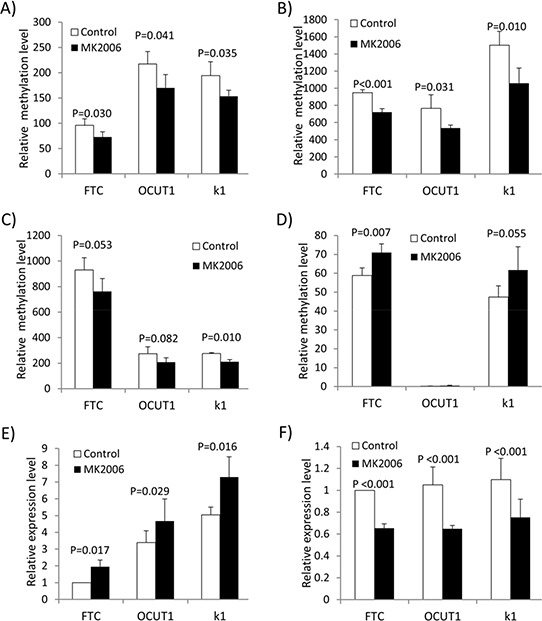
Validation of methylation microarray results by quantitative PCR **A–D.** Validation of the AKT inhibitor MK2006-induced methylation changes of *CDKN2D* (A), *REC8* (B), *GSPT1* (C) and *ADORA2B* (D) by QMSP. **E.** and **F.** Expression analysis of *REC8* (E) and *ADORA2B* (F) by qRT-PCR. Paired *T*-test was used to examine the statistical significance of the methylation or expression difference of the respective genes before and after treatment.

The ‘cell cycle process’ and ‘cellular response to extracellular stimulus process’ contain 6 hypomethylated and 3 hypermethylated genes, respectively (Figure 1D). Considering that the two processes are most associated with the biological functions of the PI3K pathway among the 9 enriched processes identified here [19], we focused on these 9 genes for further study. Quantitative methylation-specific PCR (QMSP) analysis confirmed that the methylation change in 4 of the 9 genes—*CDKN2D*, *REC8*, *GSPT1* and *ADORA2B* induced by MK2006 was consistent with the methylation array results in the three cell lines (Figures 2A–2D). The other five genes did not show consistent methylation changes between the methylation array and QMSP analyses (Supplementary Figure S1). *REC8* and *ADORA2B*, which became hypomethylated and hypermethylated, respectively, after MK2006 treatment, were correspondingly significantly up-regulated and down-regulated in their expression as detected by RT-PCR (Figures 2E and 2F; *p* < 0.05). No significant change in the expression of *CDKN2D* and *GSPT1* was observed (Supplementary Figure S2). The alteration in the methylation status of *REC8* and *ADORA2B* was thus presumptively most relevant functionally and these two genes were studied further.

### Relationship of the methylation/expression status of *REC8* and *ADORA2B* with genetic alterations of the PI3K pathway in thyroid cancer cell lines

**Figure 3:**
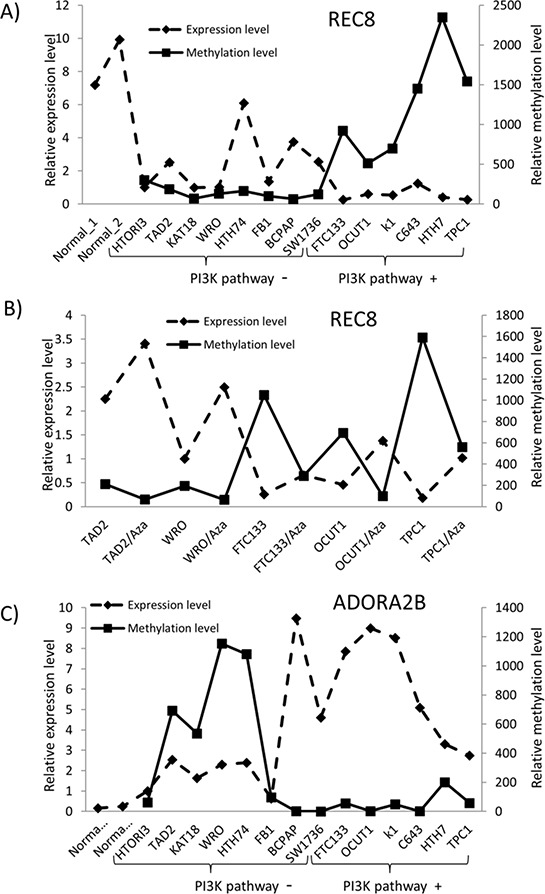
Relationship between the expression and methylation of *REC8* and *ADORA2B* and genetic alterations of PI3K/Akt pathway in thyroid cancer cell lines **A.** Methylation and expression of *REC8* in thyroid cancer cell lines with or without genetic alterations in the PI3K/AKT pathway as indicated. **B.** Effects of the demethylating agent 5-Aza-2′-deoxycytidine on the methylation and expression of *REC8.* Cells were treated with the drug (5 μM) for 72 h. **C.** Methylation and expression of *ADORA2B* in thyroid cancer cell lines with or without genetic alterations in the PI3K/AKT pathway. The relative gene expression and methylation levels were analyzed by QRT-PCR and QMSP, respectively. The left y-axis represents the relative expression level while the right axis represents the relative methylation level of the two genes. QMSP was not conducted for the two normal tissues due to the unavailability of the matched normal genomic DNA of the two samples. HTORI3 and TAD2 are two immortalized normal thyroid cell lines. The cell lines were divided into two groups, with one harboring no genetic alterations and the other harboring genetic alterations in the PI3K pathway (PI3K pathway – or PI3K pathway +, respectively), which included *PTEN* loss (FTC133), *PI3K* mutation (K1, OCUT1), *RAS* mutation (C643, Hth7) and *RET/PTC1* re-arrangement (TPC1).

To further investigate the role of the PI3K pathway in the alterations of *REC8* and *ADORA2B* genes, we examined the relationship of their methylation/expression with the genetic alterations of the PI3K pathway. We analyzed the methylation and mRNA levels of the two genes in a cell panel consisting of 14 thyroid cell lines with various genetic backgrounds of the PI3K pathway. The basal methylation level of *REC8* in the 8 cell lines harboring no genetic alterations in the PI3K pathway was significantly lower than that of the cell lines harboring genetic alterations (141.99 ± 76.52 *vs*. 1245.40 ± 676.47, *p* = 0.018), while the *REC8* mRNA level in the former cell group was significantly higher than that in the latter group (2.41 ± 1.79 *vs.* 0.55 ± 0.37, *p* = 0.031) (Figure 3A). Thus, an association between *REC8* methylation and genetic alterations in the PI3K pathway, an inverse relationship between *REC8* expression and genetic alterations in the PI3K pathway, and an inverse relationship between *REC8* expression and *REC8* methylation were observed in thyroid cancer cells. Treatment with the DNA-demethylating agent 5-Aza-2′-deoxycytidine induced promoter demethylation of *REC8*, which was otherwise normally hypermethylated by the genetically activated PI3K pathway, and restored its expression in 4 of the 5 thyroid cancer cell lines tested (Figure 3B). On the contrary, *ADORA2B* had a higher methylation level in cell lines harboring no genetic alterations in the PI3K pathway than the cells harboring genetic alterations (452.62 ± 483.89 *vs.* 61.34 ± 73.17, *p* = 0.057), while its mRNA level showed an inverse relationship between the two cell groups (3.07 ± 2.86 *vs.* 6.085 ± 2.74, *p* = 0.075) (Figure 3C). Pearson correlation coefficient (Pearson's r) was −0.473 (*p* = 0.044, one-tail) between the *REC8* expression and methylation levels and −0.463 (*p* = 0.048, one-tail) between *ADORA2B* expression and methylation levels in the 14 thyroid cells, further suggesting that DNA methylation plays an important role in the regulation of the expression of the 2 genes.

### Methylation of *REC8* and *ADORA2B* in human thyroid tumors

**Figure 4:**
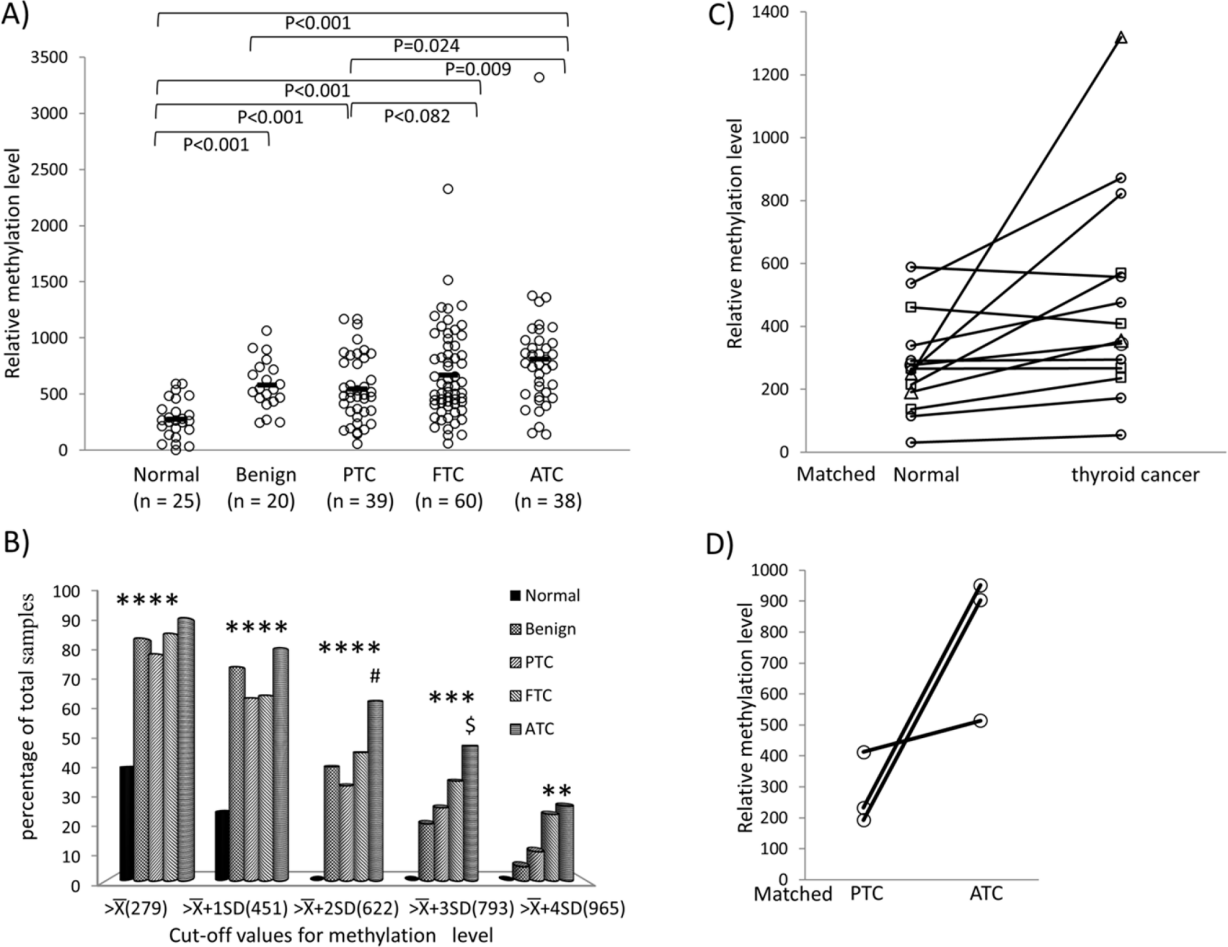
Methylation of *REC8* in various thyroid tumors—progression from low- to high-grade tumors **A.** Methylation of *REC8* in individual cases of various types of thyroid tumor, with progressive increase from low- to high-grade tumors. Each individual case of thyroid sample is represented by a circle. The average methylation level of each type of tissues is indicated with a short horizontal bar. Randomization test was used for calculation of the methylation difference between two types of thyroid samples. **B.** Prevalence of *REC8* methylation (Y-axis) at the indicated relative levels (X-axis) in different types of thyroid tumors and normal thyroid tissues. The average ($\bar{X}$) and standard deviation (SD) of the relative methylation level of *REC8* in normal thyroid tissues were 279.41 and 171.28, respectively. As indicated, the values of $\bar{X}$, $\bar{X}$+SD, $\bar{X}$+2SD, $\bar{X}$+3SD and $\bar{X}$+4SD were used to group *REC8* methylation levels. *p < 0.05 compared with normal samples, #*p* < 0.05 compared with PTC, ^$^*p* < 0.05 compared with benign tumor on Fisher's exact *T*-test. **C.** Comparison of *REC8* methylation in thyroid cancer and matched normal thyroid tissues. Each individual thyroid sample is represented by a symbol and the matched samples are connected with a black line. Circle, PTC; Rectangle, FTC; Triangle, ATC. **D.** Methylation of *REC8* in paired PTC and ATC that occurred in the same patient. Each individual patient is represented by a circle and the matched samples are connected with a line. PTC, papillary thyroid cancer; FTC, follicular thyroid cancer; ATC, anaplastic thyroid cancer.

We next examined the methylation status of *REC8* and *ADORA2B* in human thyroid tumor tissues. As shown in Figure 4A, ATC samples had the highest methylation level at 811.69 ± 524.71, followed by FTC at 672.04 ± 405.75. PTC had a lower methylation level of 547.36 ± 299.44, which was comparable with the methylation level of 582.03 ± 224.37 in benign thyroid tumors. *REC8* methylation level in each of the four tumor types was higher than the methylation level of 279.41 ± 171.28 in the normal tissue (*p* < 0.001). A similar trend of *REC8* methylation level in the tumor ranking order was observed when different cut-off values for the methylation were used, particularly at high cut-off values of $\bar{X}+3\text{SD}$ (793) and $\bar{X}+4\text{SD}$ (965). At these high levels, *REC8* methylation was a major epigenetic event mainly in ATC and FTC (Figure 4B). For example, 23.33% FTC and 26.21% ATC had *REC8* methylation higher than $\bar{X}+4\text{SD}$ (965), whereas < 10% PTC and benign tumors reached this methylation level. When all the thyroid cancers were analyzed collectively, *REC8* methylation in cancer samples was also higher than that in the matched normal thyroid tissues (Figure 4C). There were three cases of ATC tumors that had coexisting PTC components. The *REC8* methylation level in the ATC component was much higher than that in the matched coexisting PTC components (Figure 4D). No significant difference in *ADORA2B* methylation was observed among normal thyroid tissues and four types of thyroid tumor tissues (Supplementary Figure S3A). We therefore next focused on the investigation of the *REC8* gene.

### Association between *REC8* methylation and genetic alterations in the PI3K pathway in human thyroid tumors

**Figure 5:**
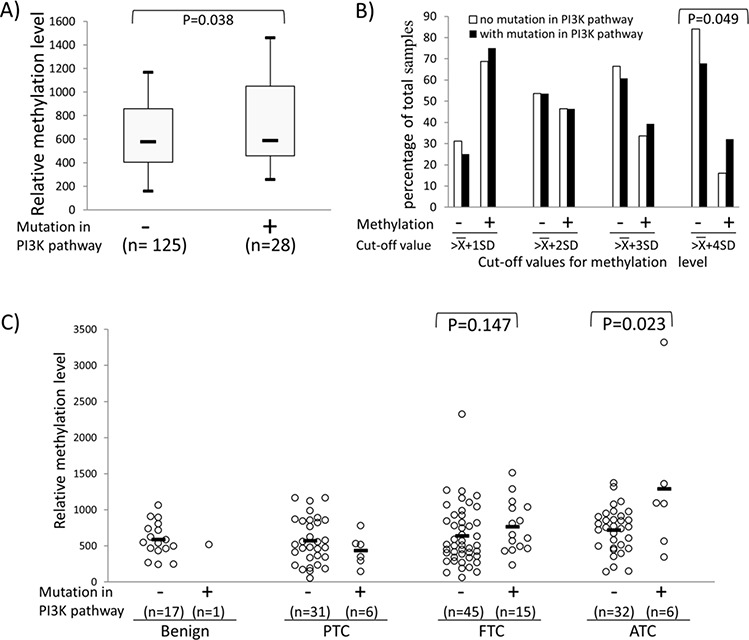
Association between *REC8* hypermethylation and genetic alterations in the PI3K/AKT pathway Genetic alterations in the PI3K/AKT pathway included any mutations in *PIK3CA*, *PTEN* and the three *RAS* genes. We successfully obtained mutation results for the 5 genes in 153 of 157 thyroid tumors tested, which were used in the analysis in this figure. **A.** *REC8* methylation levels in thyroid tumors without or withy mutations in the PI3K pathway. Shown are the Box-Whisker plots of the *REC8* methylation levels, with five statistics (lower whisker, lower box part, solid line in box, upper box part, and upper whisker presents 5th, 25th, 50th, 75th and 95th percentile, respectively). Randomization test was used to calculate the *p*-values for the difference in methylation level between tumor groups. **B.** Prevalence (Y-axis) of *REC8* methylation at the indicated cut-off levels (X-axis) in thyroid tumors with or without mutations in the PI3K pathway. The cut-off methylation values of $\bar{X}$, $\bar{X}+\text{SD}$, $\bar{X}+2\text{SD}$, $\bar{X}+3\text{SD}$ and $\bar{X}+4\text{SD}$ used are as described in the legend to Figure 4. **C.** Relationship between the *REC8* methylation and genetic alterations in the PI3K pathway in various types of thyroid tumor. Each individual case of thyroid sample is represented by a circle. Each type of tumors consisted of two groups, one with mutations and the other without mutations in the PI3K pathway as indicated. The average methylation level of each type of tissues is indicated with a short horizontal bar. Randomization test was used to examine the methylation difference between thyroid samples.

To explore further the relationship between *REC8* methylation and the PI3K pathway, we examined the hotspot mutations of five classical genes in the PI3K pathway, including *H-RAS*, *K-RAS*, *N-RAS*, *PIK3CA* and *PTEN* in 157 thyroid tumor samples (Supplementary Table S2). As shown in Figure 5A, when all the tumors were pooled for analysis, *REC8* methylation level was significantly higher in the tumors carrying any of the classical mutations (PI3K+) in the five genes than that in the tumors carrying none of the mutations (PI3K-), being 801.04 ± 610.43 in the former *vs*. 637.45 ± 347.27 in the latter (*P* = 0.038). A similar association was also observed between the *REC8* methylation and the mutations in the PI3K pathway when using different cut-off values of methylation, particularly at high values (Figure 5B). For example, 9/28 (32.14%) PI3K+ thyroid tumors versus 20/125 (16.00%) PI3K- tumors had a *REC8* methylation level > $\bar{X}+4\text{SD}$ (965) (*p* = 0.049). When analyzed in individual tumor types, PI3K+ ATC had significantly higher *REC8* methylation than that in the PI3K- ATC (1294.21 ± 1059.85 vs. 721.22 ± 306.46, *p* = 0.023) (Figure 5C). *REC8* also showed higher methylation in PI3K+ FTC than in PI3K- FTC, although the difference did not reach statistical significance. No correlation between *ADORA2B* methylation and mutations in the PI3K pathway was observed (Supplementary Figure S3B).

### Replication of the associative relationship between *REC8* methylation/expression and genetic alterations/signaling activities of the PI3K pathway in cancer genome atlas (TCGA) database

**Figure 6:**
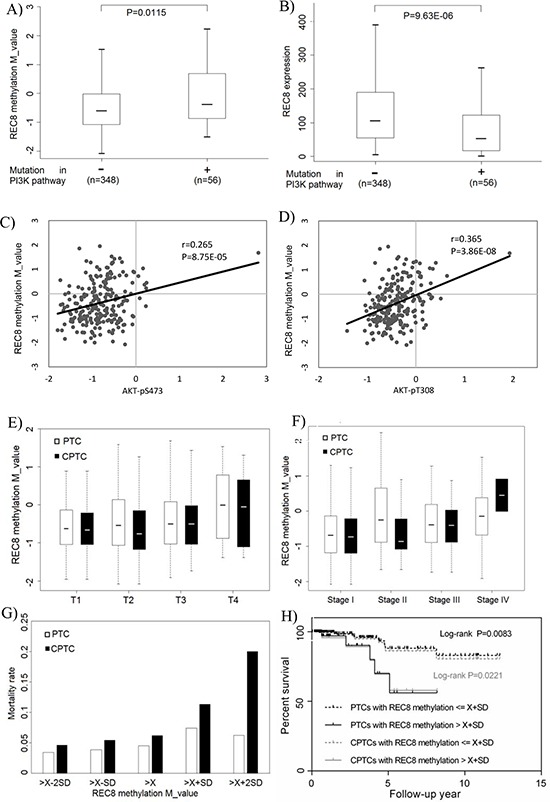
Association of *REC8* hypermethylation with genetic alterations/signaling activities of the PI3K pathway and poor clinical outcomes of thyroid cancer in TCGA thyroid cancer database **A.** Association between *REC8* hypermethylation and genetic alterations in the PI3K pathway in thyroid cancer. The genetic alterations included mutations in *PIK3CA, PTEN* and the three *RAS* genes. **B.** Inverse association between the *REC8* mRNA expression and the above genetic alterations in the PI3K pathway. **C.** Association between *REC8* hypermethylation and AKT-pS473 phosphorylation. **D.** Association between *REC8* hypermethylation and AKT-pT308 phosphorylation. **E.** Association between REC8 hypermethylation and high tumor stages (T, on the TNM classification). REC8 methylation was T1 = −0.626 ± 0.721 vs T4 = −0.008 ± 0.982 (*P* = 0.047) for all PTC and T1 = −0.663 ± 0.752 vs T4 = −0.051 ± 0.921 (*P* = 0.087) for conventional PTC (CPTC). **F.** Association between REC8 methylation and thyroid cancer stages on the American Joint Commission of Cancer staging system. Comparison of REC8 methylation in stages II (−0.688 ± 0.771), III (−0.384 ± 0.708) and IV (−0,141 ± 0.826) with that in stage I (−0.688 ± 0.771) showed *P* = 0.002434, 0.002224, and 0.000651, respectively, on the analysis of all PTC. Comparison of *REC8* methylation in stage III (−0.400 ± 0.650) and stage IV (−0.452 ± 0.650) with stage I (−0.727 ± 0.740) showed *P* = 0.010 and < 0.001, respectively, on the analysis of CPTC. **G.** Association between *REC8* hypermethylation and thyroid cancer-related patient mortality. Mortality rates (Y-axis) are shown for the indicated *REC8* methylation levels (X-axis) in terms of average ($\bar{X}$) and standard deviation (SD) of M values of *REC8* methylation, which were −0,453 and 0.813 for all PTC patients and −0.542 and 0.761 for CPTC patients, respectively. REC8 methylation levels were grouped as $\bar{X}-2\text{SD}$, $\bar{X}-\text{SD}$, $\bar{X}$, $\bar{X}+\text{SD}$ and $\bar{X}+2\text{SD}$, with the mortality rate progressively ring from low to high methylation levels. **H.** Kaplan-Meier survival curves by the REC8 methylation levels > $\bar{X}+\text{SD}$ and ≤ $\bar{X}+\text{SD}$. Comparison of the two groups resulted in *P* = 0.0083 on the analysis of all PTC and *P* = 0.0221 on the analysis of CPTC.

Although the above data showed that compared with FTC and ATC, fewer cases of PTC harbored *REC8* methylation, we speculated that the large number of PTC tumors in the TCGA database could be sufficient for a replicative study of our findings on *REC8*. By analyzing thyroid cancer data retrieved from the TCGA database, we indeed demonstrated an association between *REC8* hypermethylation and genetic alterations in the PI3K pathway (Figure 6A) and an inverse association between the *REC8* mRNA expression and the genetic alterations in the PI3K pathway (Figure 6B). We additionally demonstrated an association between *REC8* hypermethylation and increased PI3K signaling activities in terms of AKT phosphorylation, including AKT-pS473 (Figure 6C) and AKT-pT308 (Figure 6D). No mutation was found on our sequencing of the exons of *REC8* in thyroid cancer or on the analysis of the TCGA thyroid cancer database, suggesting that mutation of this gene is not a common event in thyroid cancer.

Analysis of the TCGA data also revealed hypermethylation of the *REC8* gene in many other human cancers (Supplementary Figure S4). Similar relationship between *REC8* and the PI3K pathway was also seen in some other cancers. For example, we found a significant association between *REC8* hypermethylation and genetic alterations in the PI3K pathway (Supplementary Figure S5A) and a trend of inverse association between the *REC8* mRNA expression and genetic alterations in the PI3K pathway (Supplementary Figure S5B) in cutaneous melanoma. There was also a significant association between *REC8* hypermethylation and AKT-pS473 phosphorylation (Supplementary Figure S5C) and AKT- pT3083 phosphorylation (Supplementary Figure S5D) in primary melanoma. Virtually identical results were found with glioma (Supplementary Figure S6).

Gene expression signature derived from defined experimental perturbation of a given signaling activity is now a widely used measure for the pathway activity as the signature offers the advantage of providing a measure of pathway activity irrespective of how the pathway may have been activated [20]. By applying the PI3K pathway signature to 2 microarray datasets that comprise 566 colon cancer samples [21] and 180 melanoma samples [22], respectively, we obtained the PI3K pathway activity values for each of the 746 cancer samples (Supplementary Table S3). REC8 expression level showed again an inverse relationship with the PI3K activities in colon cancer (Supplementary Figure S7A) and melanoma (Supplementary Figure S7B).

**Figure 7:**
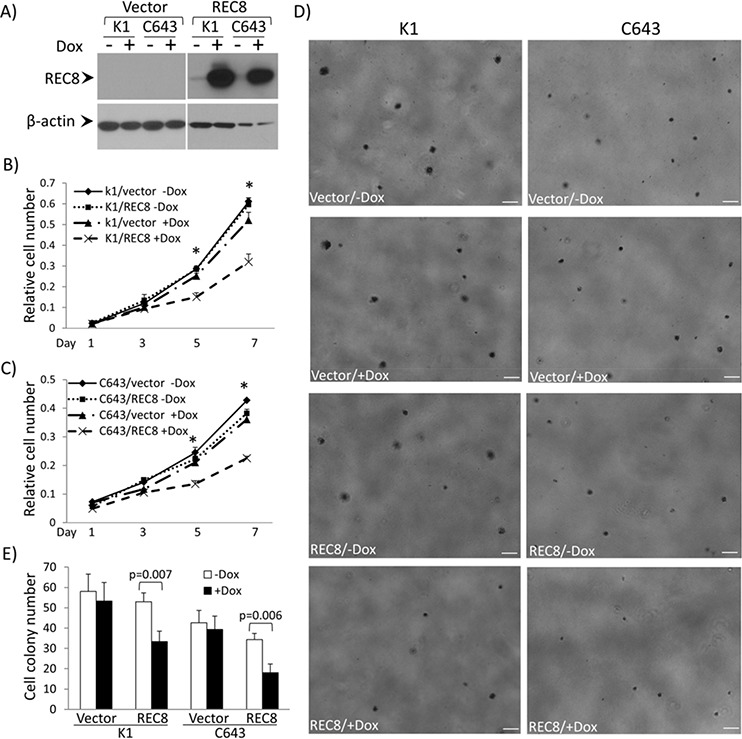
Examination of tumor cell-suppressing functions of the *REC8* in thyroid cancer cells **A.** Western blotting analysis of inducible expression of *REC8* in thyroid cancer cell lines K1 and C643. The procedures for the establishment of doxycycline (Dox)-inducible expression of *REC8* in K1 cells and C643 are as described in the Supplementary Materials and Methods. **B.** Effects of Dox-induced expression of *REC8* on the proliferation of K1 cells. **C.** Effects of Dox-induced expression of *REC8* on the proliferation of C643 cells. Cell proliferation was analyzed using MTT assay, in which the OD value was measured every 2 days over a 7-day time course. Results are expressed as means ± SD, *n* = 4. *T*-test was used to compare the cell numbers at day 5 and 7 respectively. **D.** Effects of Dox-induced expression of *REC8* on the colony formation of K1 and C643 cells. Scale bar = 200 μm. **E.** Bar graph presentation of cell colony numbers from 3 experiments (mean ± SD) corresponding to D). *On *T* test, *p* < 0.01.

### Association between *REC8* hypermethylation and poor clinicopathological outcomes of PTC in the TCGA database

On the analysis of the TCGA database, we observed an association between *REC8* hypermethylation and high tumor stages (T) (Figure 6E). For example, *REC8* methylation in T4 tumors was significantly higher than that in T1 tumors on the analysis of all PTC (−0.008 ± 0.982 in T4 vs −0.626 ± 0.721 in T1, *p* = 0.047), with a trend on the analysis of conventional type of PTC (CPTC) alone, the most common variant of PTC (−0.051 ± 0.921 in T4 vs −0.663 ± 0.752 in T1, *p* = 0.087). A similar association was also observed between *REC8* methylation and thyroid cancer stages on the American Joint Commission of Cancer staging system (Figure 6F). Specifically, on the analysis of all PTC patients, *REC8* methylation in stage II (−0.25 ± 0.921), stage III (−0.384 ± 0.708), and stage IV (−0,141 ± 0.826) patients was all significantly higher than that in stage I patients (−0.688 ± 0.771) (*p* = 0.002, 0.002, and < 0.001, respectively). On the analysis of CPTC patients alone, *REC8* methylation in stage III (−0.400 ± 0.650) and stage IV (0.452 ± 0.650) patients was significantly higher than that in stage I patients (−0.727 ± 0.740) (*p* = 0.01 and < 0.001, respectively). We also observed an association between *REC8* hypermethylation and thyroid cancer-related patient mortality (Figure 6G). Specifically, on grouping the patients based on *REC8* methylation levels of $\bar{X}-2\text{SD}$, $\bar{X}-\text{SD}$, $\bar{X}$, $\bar{X}+\text{SD}$ and $\bar{X}+2\text{SD}$, a strong rising trend of mortality from low to high methylation levels was revealed, most prominently in the patients with methylation > $\bar{X}+\text{SD}$. A significantly higher mortality rate was observed in the patients with methylation > $\bar{X}+\text{SD}$ than that in the patients with methylation ≤ $\bar{X}+\text{SD}$ on the analysis of all PTC (*P* = 0.0523) or CPTC alone ((*P* = 0.035). Kaplan-Meier analysis showed that patients with higher *REC8* methylation level (>$\bar{X}+\text{SD}$) had a significantly lower survival probability than patients with lower methylation level (≤X+SD) on the analysis of either all PTC (*p* = 0.008) or CPTC alone (*p* = 0.022) (Figure 6H).

### Inhibition of thyroid cancer cell by REC8

To functionally test a role of *REC8* in thyroid tumorigenesis, we constructed REC8 expression vector and established stable transfectant thyroid cancer cell lines K1 and C643 with doxycycline-inducible expression of REC8 (Figure 7A). Cell proliferation assay over a 7-day time course showed that both K1 and C643 cells with induced *REC8* expression showed a significantly lower proliferation rate than cells transfected with the empty vector or *REC8*-transfected cells without induction (Figures 7B and 7C). Significant difference in cell number occurred on day 5 for both K1 and C643 cells (*p* < 0.01). On day 7, the difference was more pronounced; expression of REC8 inhibited K1 cell proliferation to 61.42% and C643 proliferation to 62.43%. REC8 expression also significantly inhibited the colony formation of K1 and C643 cells (Figures 7D and 7E); the colony numbers before and after induced REC8 expression were 53.33 ± 9.07 *vs*. 33.33 ± 5.13 for K1 cells and 39.33 ± 6.51 *vs*. 18.00 ± 4.36 for C643 cells (for both comparisons, *p* < 0.001). The colonies also became smaller upon induced REC8 expression.

## DISCUSSION

As in the tumorigenesis of many other human cancers [23], aberrantly activated PI3K pathway driven by its mutations is a fundamental mechanism in thyroid tumorigenesis [6, 9]. Inspired by our recent demonstration that genome-wide alterations in DNA methylation and resulting aberrant activities of cancer-related genes coupled to the aberrantly activated MAP kinase pathway is a prominent mechanism in the tumorigenesis of PTC [16], in the present study we hypothesized that a similar mechanism existed in thyroid tumorigenesis driven by the PI3K pathway. Indeed, using the Illumina Methylation 450K array system we showed here that many genes became hypo- or hyper-methylated upon suppression of the PI3K pathway in thyroid cancer cells, thus demonstrating an epigenetic coupling of the PI3K pathway to many genes with a wide range of functions in the genome in thyroid cancer. As many of these genes epigenetically targeted by the PI3K pathway may be oncogenetically important, their aberrant changes in expression, through alterations in their methylation by the PI3K pathway, are expectedly important tumor-promoting events. This represents a major molecular mechanism in thyroid tumorigenesis driven by the PI3K pathway. With our strategy using strict criteria to explore genes with the most robust changes in methylation and expression and with the highest functional potential as a cancer gene on the Gene Ontology analysis, we identified *REC8* as a particularly promising novel cancer-related gene. The results in our cell lines and thyroid tumors on the link between REC8 and the PI3K pathway were robust and fully replicated in the TCGA database. *REC8* hypermethylation was also strongly associated with poor clinicopathological outcomes of thyroid cancer patients, including advanced tumor and disease stages and patient mortality. Our functional studies also directly demonstrated the inhibitory effects of REC8 expression on thyroid cancer cells. These results establish that *REC8* is a prominent novel tumor suppressor gene that is epigenetically robustly targeted by the PI3K pathway in thyroid cancer.

REC8 is classically known to be a key component of the meiotic cohesion complex that plays a critical role in chromosome dynamics during meiosis, including homology chromosome pairing, crossover recombination, and sister chromatid cohesion during meiosis [24, 25]. In this context, it makes good sense to see *REC8* as a tumor suppressor gene as, from an evolutionary perspective, expression of *REC8* in germ cells is a protective and surviving mechanism that prevents oncogenesis early in life so heathy germ cells can normally reproduce and life can continue. REC8 is not only expressed in gonad tissues as a meiotic gene, but also expressed in normal non-gonadal somatic tissues, suggesting that *REC8* may have other undefined important biological functions. This was indeed demonstrated to be the case in the present study.

It is interesting to see that hypermethylation of the *REC8* gene was uniquely more common in FTC and ATC, particularly when looking at high methylation levels. Methylation of *REC8* was highly associated with the classical mutations that drive the PI3K pathway both in thyroid cancer cell lines and in thyroid cancers. For the latter, this was also particularly the case with FTC and ATC. These data are well consistent with the fact that the PI3K pathway plays a particularly important role in FTC and ATC and genetic alterations in the PI3K pathway are most common in these two tumors among all types of thyroid tumors [6, 9]. These data further support that *REC8* is a prominent epigenetic target of the PI3K pathway and its silencing through aberrant methylation plays a major role in mediating the oncogenic function of the PI3K pathway in thyroid tumorigenesis.

The novel role of *REC8* as a tumor suppressor gene identified in the present study is consistent with previous findings that sister chromatid cohesion has a crucial role in double-strand break repair of DNA [24] and genotoxic insult could induce the expression of *REC8* [26, 27], suggesting that *REC8* might play an important role in DNA damage repair and maintenance of chromosome stability. Previous studies showed that *REC8* was hypermethylated in melanoma [28, 29, 29, 30] and malignant gastrointestinal stromal tumor [28, 29, 29, 30] and, in the latter, hypermethylation of REC8 was associated with a poorer prognosis of the tumor. It is worth noting that, among all types of thyroid cancers, hypermethylation of *REC8* most prominently occurred in ATC, the most aggressive type of thyroid cancer, and was associated with advanced disease stages of thyroid cancer and thyroid cancer-related mortality. Our analysis of the TCGA data also showed hypermethylation and silencing of *REC8* in many other human cancers. A similar relationship between *REC8* and the PI3K pathway was seen in several other cancers. Therefore, the data strongly suggest that *REC8* is a *bona fide* tumor suppressor gene and its epigenetic inactivation by the PI3K pathway may play an important role widely in human tumorigenesis driven by the PI3K pathway. As the PI3K pathway can be activated by protein stimuli in the extracellular microenvironments, the nuclear division regulation by REC8 as the downstream molecular of PI3K pathway might be potentially linked to the extracellular microenvironment homeostasis; the latter is an important mechanism in thyroid tumorigenesis [31].

It should be noted that although *REC8* was specifically investigated here as a gene that was robustly controlled epigenetically by the PI3K pathway, there are other important genes epigenetically regulated by the PI3K pathway that could also be important cancer-related genes (e.g., some of the genes in Supplementary Table S1). These may potentially include novel oncogenes as well. Thus, there is considerable room to explore and identify additional important novel cancer-related genes epigenetically controlled by the PI3K pathway in future studies.

It is not clear how methylation of *REC8* is regulated by the PI3K pathway. It was previously shown that PI3K pathway could regulate the methylation of histone through phosphorylation of EZH2 [32–34], the expression of DNA methyltransferase DNMT3A [35], and the stability of DNA methyltransferase DNMT1 [36]. While these could all be involved in the epigenetic regulation of *REC8* by the PI3K pathway, specific and fine micro environmental regulations of the gene are presumably important molecular events in the targeted epigenetic regulation of the *REC8* gene by the PI3K pathway. A such example is the methylation and silencing of the *Cystatin M* (CST6) gene by the PI3K pathway [37], in which histone h3k27me3 and h3k9me2, which recruit the DNA methyltransferase for *de novo* methylation of DNA [38, 39], were significantly enriched in the *CST6* promoter and its surrounding regions upon activation of the PI3K pathway [37]. This is likely a mechanism involved in the PI3K pathway-promoted *REC8* methylation in human cancers.

In summary, we for the first time demonstrate genome-wide aberrant methylation alterations of genes by the PI3K pathway in thyroid cancer and identify specifically *REC8* as a novel tumor suppressor gene that, through robust epigenetic inactivation by the PI3K pathway, plays an important and wide role in thyroid cancer and other human cancers. This study thus uncovers an important novel mechanism mediating the oncogenic function of the PI3K pathway in human tumorigenesis, as exemplified by thyroid tumorigenesis. The study also demonstrates a prognostic value of *REC8* hypermethylation for poor prognosis of thyroid cancer and potentially other cancers as well.

## MATERIALS AND METHODS

### Thyroid cancer cell line culture

The thyroid cancer cell lines were all grown at 37°C in RPMI 1640 medium with 10% fetal bovine serum (FBS), except for FTC133 which was cultured in DMEM/HAM'S F-12 medium. The procedures were performed following standard cell culture protocols.

### Methylation microarray and data analysis

Bisulfite-converted DNA was analyzed for the methylation status using Illumina's Infinium Human Methylation 450 array. The statistical analysis was performed in R (version 2.15) using packages from the Bioconductor projects [40]. The lumi package, which is specifically designed for Illumina data, was used to calculate M-value, the log2 ratios of methylated over unmethylated normalized signal intensities [41]. The methylation microarray data output from GenomeStudio was quantile color balance-adjusted, quantile normalized and then converted to the M values. Only probes located in transcriptional regulatory regions, including TSS1500, TSS200, 5′-UTR, enhancer and 1st exon regions were analyzed in the current study. Univariate paired *T*-test was used to identify CpG sites with significant methylation changes induced by MK2006 in thyroid cancer cells. The differentially methylated CpG sites were mapped to corresponding genes according to the gene annotation data provided by the array manufacturer. To ensure both high statistical significance and strong biological effects, we require that differentially methylated CpG sites had a *p*-value < 0.005 and fold change (based on M-value) > 1.5 in each of three cell lines in the same direction. Gene Ontology (biological process terms level 3) enrichment was conducted using the DAVID functional annotation tool (http://david.abcc.ncifcrf.gov) with default parameters.

### Prediction of PI3K pathway activities based on the microarray gene expression profile

The raw microarray data used for this purpose were downloaded from Gene Expression Omnibus (GEO) and normalized by Robust Multi-array Average (RMA) approach in R environment (http://www.r-project.org). The Bayesian binary regression algorithm was used to analyze the microarray gene expression data and predict PI3K pathway activity [20].

### The TCGA data analysis

Related whole-exome somatic mutation data, DNA methylation data, mRNAseq data and the clinical data were obtained from the TCGA Data Portal (https://tcga-data.nci.nih.gov/tcga/). For somatic mutation analysis, the mutation information annotated with “Silent” or “Intron” was excluded from our analysis for their presumed lack of immediate functional consequences. We used M-values to perform the methylation analysis, which involved downloading of the level 1 data for M-values calculation. A total of 399 thyroid cancer samples with complete mutation, methylation and clinical data were analyzed.

### Statistical analysis

Chi-square test and Fisher's exact *T*-test were used for categorical data when the numbers in the cells for cross-tabulation were >5 and ≤5, respectively. The reported *P* values were two-sided and *p* < 0.05 was considered statistically significant. For continuous data, *T*-test and randomization test were used when the sample variances are equal and unequal, respectively. The randomization test was conducted as we described previously [42]. Pearson correlation coefficient r and the corresponding *p*-value were calculated for the analysis of the relationship between *REC8* expression/methylation and PI3K pathway activities. Kaplan-Meier survival curve with log-rank test, censoring patients at the time of last follow-up, was used to compare the patient survival rates by the *REC8* methylation level.

Additional details of the methodology are described in the online Supplementary Materials and Methods.

## SUPPLEMENTARY FIGURES AND TABLES








