# Supplementary material for: REC8 is a novel tumor suppressor gene epigenetically robustly targeted by the PI3K pathway in thyroid cancer
Source: Oncotarget. 2015 Oct 13;6(36):39211–24. doi: 10.18632/oncotarget.5391 (PMC4770767; doi:10.18632/oncotarget.5391)
Supplement: Supplementary file 1 [file oncotarget-06-39211-s001.pdf]

SUPPLEMENTARY MATERIALS AND METHODS

Thyroid cancer cell lines

The thyroid cancer cell lines C643, Hth7, Hth74, and SW1736 were originally from Dr. N.E. Heldin (University of Uppsala, Uppsala, Sweden); KAT18 from Dr. Kenneth B. Ain (University of Kentucky Medical Center, Lexington, KY); OCUT1 from Dr. Naoyoshi Onoda (Osaka City University Graduate School of Medicine, Osaka, Japan); BCPAP from Dr. Massimo Santoro (University of Federico II, Naples, Italy); K1 from Dr. David Wynford-Thomas (University of Wales College of Medicine, Cardiff, UK); WRO-82-1 from Dr. G. J. F. Juillard (University of California-Los Angeles School of Medicine, Los Angeles, CA); and FTC133 from Dr. Georg Brabant (University of Manchester, Manchester, UK). The normal thyroid cell-derived cell line TAD2 was from Dr. Mario Vitale (Università Federico II, Naples, Italy). The TPC1 cell line was provided by Dr. Alan P Dackiw (Johns Hopkins University, Maryland). They were all grown at 37°C in RPMI 1640 medium with 10% fetal bovine serum (FBS), except for FTC133 that was cultured with DMEM/HAM'S F-12 medium. For some experiments, cells were treated with 0.5 μM Akt inhibitor MK2006 (ChemieTek, Indianapolis, IN) or 5 μM 5-Aza-2′-deoxycytidine (Sigma, St. Louis, MO) for 72 h with the drug being replenished every 24 h.

Sodium bisulfite conversion

DNA bisulfite conversion was carried out using EZ DNA Methylation Kit (Zymo Research, Irvine, CA) by following manufacturer's manual with minor modifications. Briefly, 0.5 – 1.0 μg of genomic DNA was first mixed with 5 μl of M-Dilution Buffer and incubate at 37°C for 15 minutes and then mixed with 100 μl of CT Conversion Reagent prepared as instructed in the kit's manual. Mixtures were incubated in a thermocycler with 16 thermal cycles at 95°C for 30 seconds and 50°C for one hour. Bisulfite-converted DNA samples were loaded onto 96-column plates provided in the kit for desulphonation and purification.

Infinium chip assay

Bisulfite-converted DNA was analyzed using Illumina's Infinium Human Methylation450 array (WG-314-1001) that allows the interrogation of > 450,000 CpG sites across the entire genome covering 99% of RefSeq genes. The BeadChips were scanned with an iScan and then analyzed by the GenomeStudio software. This experiment was conducted at The Sidney Kimmel Cancer Center Microarray Core Facility at Johns Hopkins University.

Quantitative methylation-specific PCR (QMSP)

To validate the methylation level of candidate genes identified by methylation microarray, SYBR-green-based QMSP PCR was conducted in an ABI Prism 7900HT Sequence Detector (Applied Biosystems). Normal leukocyte DNA was methylated *in vitro* with Sss I methylase (New England Biolabs) to generate completely methylated DNA as a positive control for all genes. Each plate included multiple water blanks and serial dilutions of the positive control for constructing the calibration curve on each plate. The relative degree of methylation of each DNA sample was calculated using the method as we described previously [1]. The primer sequences are presented in Supplementary Table S4.

RNA extraction and real-time quantitative RT-PCR (qRT-PCR) analysis

Total RNA was isolated using TRIzol reagent according to the instructions of the manufacturer (Invitrogen, Grand Island, NY), following by reverse-transcription using Oligo-dT and SuperScript II according to the instructions of the manufacturer (SuperScript First-Strand Synthesis kit, Invitrogen, Grand Island, NY). SYBR Green based-Realtime quantitative RT-PCR analysis was carried out in an ABI Prism 7900HT Sequence Detector (Applied Biosystems, Grand Island, NY), using SYBR Green Supermix (Bio-Rad, Hercules, CA). The expression value of each gene was normalized to β-actin cDNA to determine the relative level of RNA in each sample using the $2^{-\Delta\Delta Ct}$ method. The two normal human thyroid RNA samples used as the control were purchased from Stratagene (La Jolla,CA) and Clontech (Mountain View, CA) respectively. The primer sequences are presented in Supplementary Table S4.

Gene expression microarray datasets

The raw microarray data of all the datasets used in this study were downloaded from Gene Expression Omnibus (GEO) and normalized by Robust Multi-array Average (RMA) approach in R environment (http://www.r-project.org). To generate the merged melanoma dataset, 5 microarray datasets (GSE10282[2], GSE10916 [3], GSE15605 [4], GSE22787 [5] and GSE33728 [6]) were normalized by RNA approach, and the gene expression data of these 5 datasets were then merged using ComBat program to remove dataset-specific biases as we described previously [7]. Principal component analysis was used to check whether the dataset-specific biases were successfully removed.

Prediction of PI3K/Akt pathway activity basing on the microarray data

The prediction model for PI3K pathway activity, which is basing on gene expression profile in Affymetrix U133 arrays and includes the information of regression coefficients of each PI3K-signature gene and the intercept values of the model, were derived from reference [8]. Prediction of the pathway activity basing on the microarray gene expression data by the Bayesian binary regression algorithm was conducted as we previously described [7]. Briefly, PI3K pathway scores were firstly calculated by adding up the products of the gene expression for each signature gene and its corresponding regression coefficient, and then were scaled using the intercept values provided in the PI3K pathway prediction model and standardized by median centering (standard deviation was set as 1).

Plasmids and cell transfection

We recently constructed a *RASAL1* inducible expression plasmid basing on a modified plenti6/V5-DEST (Invitrogen, Grand Island, NY) [9]. To construct *REC8* inducible expression plasmid, we replaced the *RASAL1* cDNA in the modified lentivirus plasmid with *REC8* cDNA that was tagged with c-myc epitope (Origene, Rockville, MD). To generate lentiviral particles, human embryonic kidey 293 cells (ATCC, Manassas, VA, USA) were co-transfected with the lentiviral vector and compatible packaging plasmid mixture using Lipofectamine 2000 (Invitrogen, Carlsbad, CA), and the supernatant containing lentivirus was collected 48 h after transfection. For virus infection, cells were exposed to lentivirus-containing supernatant for 24 hours in the presence of Polybrene (Sigma, St. Louis, MO). After selection with blasticidin for 2 weeks, stable cell pools were used for cell proliferation and colony formation studies.

Western blot analysis

Cells were lysed in the RIPA buffer supplemented with phosphatase and protease inhibitors (Sigma, St. Louis, MO) and protein blot analyses were performed as previously described [10]. The antibodies used in the present study, including anti-phospho-Akt (Sc-7985-R), anti-myc tag (sc-47694) and anti-actin (Sc-1616-R), were purchased from Santa Cruz Biotechnology (Santa Cruz, CA).

Cell proliferation assay

Cells (800/well) were seeded into 96-well plates. For MTT assay, cell culture was added with 10 μl of 5 mg/ml MTT agent (Sigma St. Louis, MO) and incubated for 4 h, followed by addition of 100 μl of 10% SDS solution and a further incubation overnight. The plates were then read on a microplate reader using the test wavelength of 570 nm and the reference wavelength of 670 nm. Four duplicates were done to determine each data point. MTT assay was performed every two days over a 7-day time course to evaluate cell proliferation.

Colony formation assay

Colony formation assays were performed as described previously [11]. Briefly, cells (5×10^3 cells/well) were plated in RPMI 1640 containing 10% FBS and 0.33% agar in 6-well plates. After 3–4 weeks of culture, colonies were photographed and colony number was counted under a microscope.

REFERENCES

1. Hu S, Liu D, Tufano RP, Carson KA, Rosenbaum E, Cohen Y, Holt EH, Kiseljak-Vassiliades K, Rhoden KJ, Tolaney S, Condouris S, Tallini G, Westra WH, et al. Association of aberrant methylation of tumor suppressor genes with tumor aggressiveness and BRAF mutation in papillary thyroid cancer. Int J Cancer 2006; 119:2322–2329.
2. Augustine CK, Jung SH, Sohn I, Yoo JS, Yoshimoto Y, Olson JA, Jr., Friedman HS, Ali-Osman F, Tyler DS. Gene expression signatures as a guide to treatment strategies for in-transit metastatic melanoma. Mol Cancer Ther 2010; 9:779–790.
3. Augustine CK, Yoo JS, Potti A, Yoshimoto Y, Zipfel PA, Friedman HS, Nevins JR, Ali-Osman F, Tyler DS. Genomic and molecular profiling predicts response to temozolomide in melanoma. Clin Cancer Res 2009; 15:502–510.
4. Raskin L, Fullen DR, Giordano TJ, Thomas DG, Frohm ML, Cha KB, Ahn J, Mukherjee B, Johnson TM, Gruber SB. Transcriptome Profiling Identifies HMGA2 as a Biomarker of Melanoma Progression and Prognosis. J Invest Dermatol 2013; 10.
5. Bloethner S, Chen B, Hemminki K, Muller-Berghaus J, Ugurel S, Schadendorf D, Kumar R. Effect of common B-RAF and N-RAS mutations on global gene expression in melanoma cell lines. Carcinogenesis 2005; 26:1224–1232.
6. Widmer DS, Cheng PF, Eichhoff OM, Belloni BC, Zipser MC, Schlegel NC, Javelaud D, Mauviel A, Dummer R, Hoek KS. Systematic classification of melanoma cells by phenotype-specific gene expression mapping. Pigment Cell Melanoma Res 2012; 25:343–353.
7. Liu D, Liu X, Xing M. Activities of multiple cancer-related pathways are associated with BRAF mutation and predict the resistance to BRAF/MEK inhibitors in melanoma cells. Cell Cycle 2013; 13.
8. Gatza ML, Lucas JE, Barry WT, Kim JW, Wang Q, Crawford MD, Datto MB, Kelley M, Mathey-Prevot B,

Potti A, Nevins JR. A pathway-based classification of human breast cancer. Proc Natl Acad Sci U S A 2010; 107:6994–6999.

9. Liu D, Yang C, Bojdani E, Murugan AK, Xing M. Identification of RASAL1 as a Major Tumor Suppressor Gene in Thyroid Cancer. J Natl Cancer Inst 2013; 105:1617–1627.

10. Liu D, Hou P, Liu Z, Wu G, Xing M. Genetic alterations in the phosphoinositide 3-kinase/Akt signaling pathway confer sensitivity of thyroid cancer cells to therapeutic targeting of Akt and mammalian target of rapamycin. Cancer Res 2009; 69:7311–7319.

11. Liu D, Liu Z, Condouris S, Xing M. BRAF V600E maintains proliferation, transformation, and tumorigenicity of BRAF-mutant papillary thyroid cancer cells. J Clin Endocrinol Metab 2007; 92:2264–2271.

SUPPLEMENTARY FIGURES AND TABLES

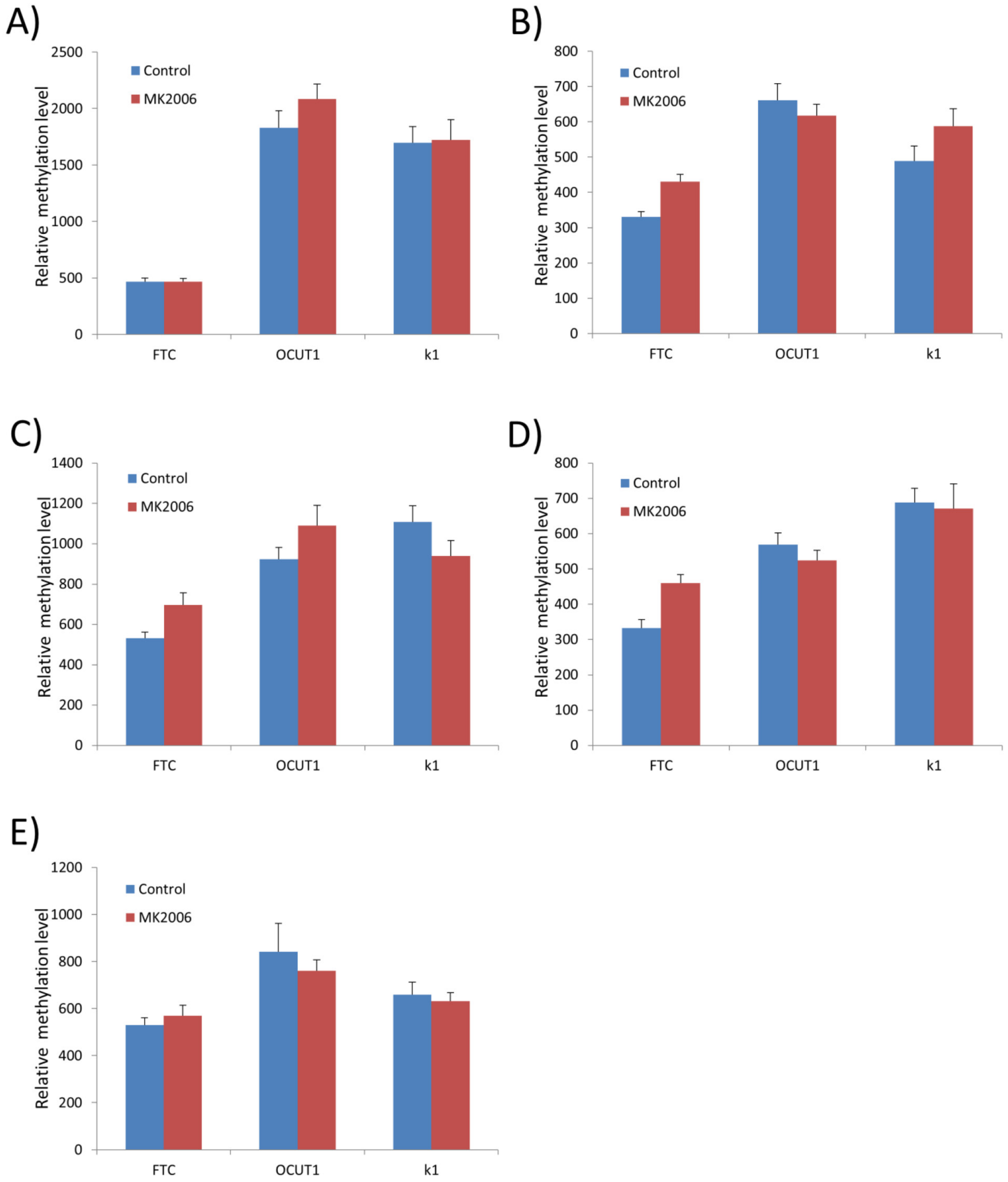

Supplementary Figure S1: Validation of methylation microarray results by quantitative methylation specific PCR for candidate genes. A. *ATG4C*; **B.** *CCNA1*; **C.** *PSMD6*; **D.** *RPTOR*; **E.** *TCF7L2*. Cells were treated with 1 μM AKT inhibitor MK2006 for 72 h with the drug being replenished every 24 h.

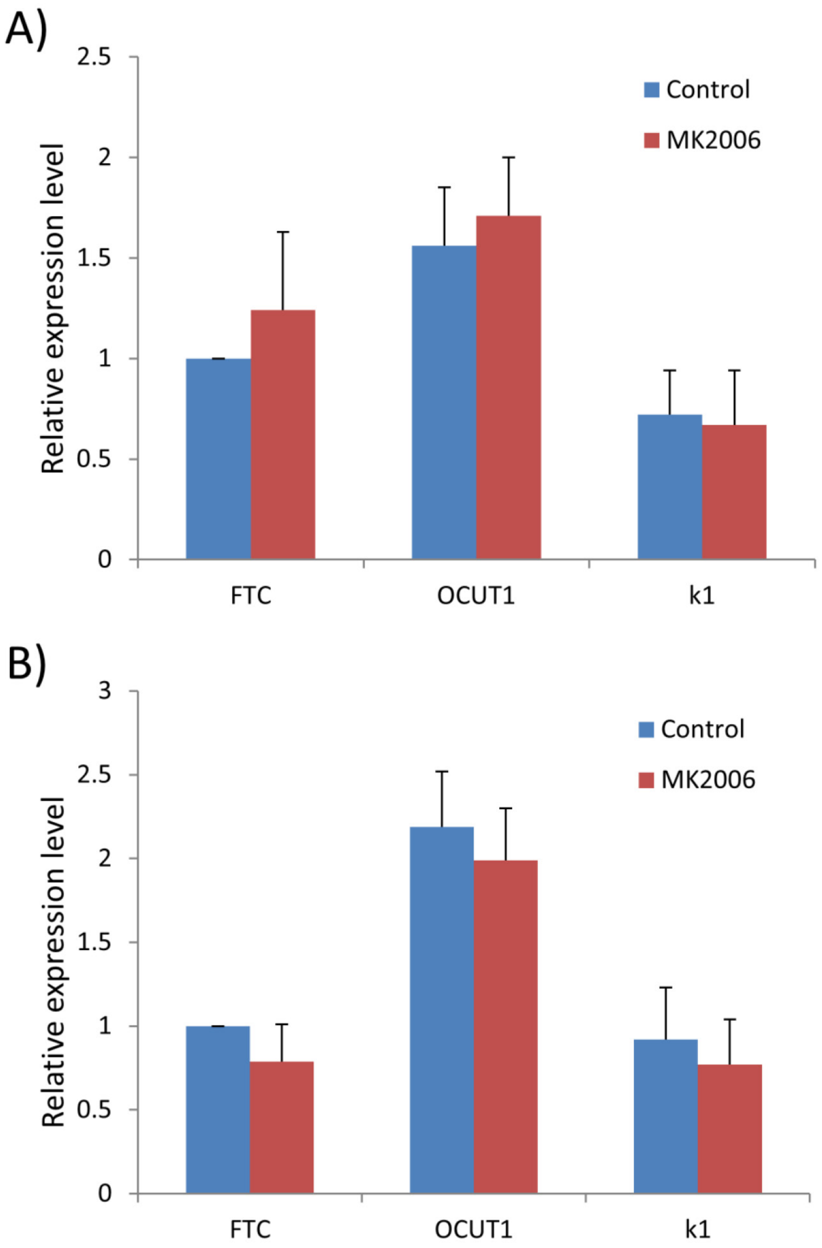

Supplementary Figure S2: Quantitative RT-PCR analysis for the expression of candidate genes in thyroid cancer cells before and after MK2006 treatment. A. *CDKN2D*; **B.** *GSPT1*. Cells were treated with 1 µM AKT inhibitor MK2006 for 72 h with the drug being replenished every 24 h.

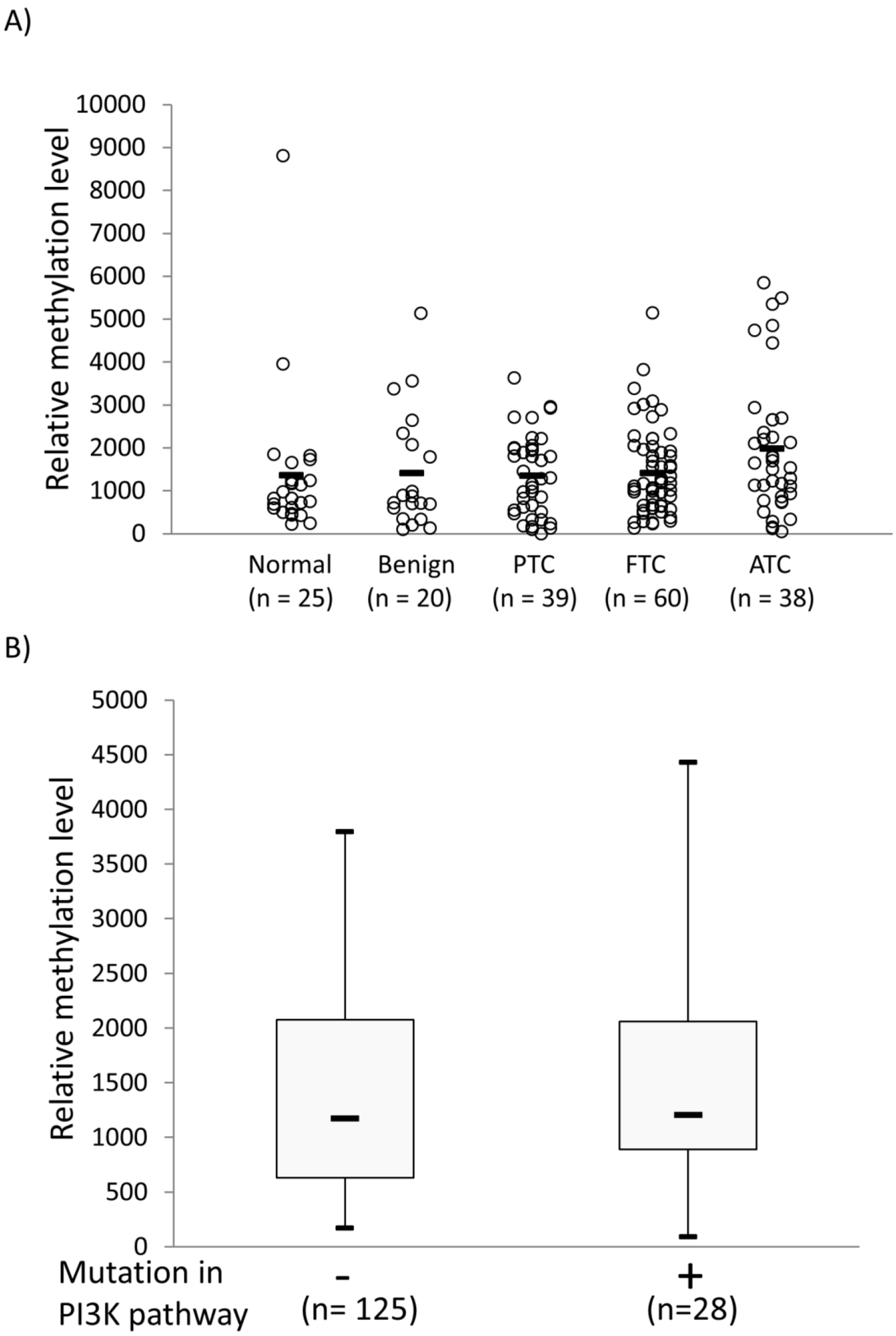

Supplementary Figure S3: Methylation of *ADORA2B* in thyroid tumors and its relationship with mutations in the PI3K pathway. A. Methylation of *ADORA2B* in individual cases of various types of thyroid tumor. Each individual case of thyroid sample is represented by a circle. The average methylation level of each type of tissues is indicated with a short horizontal bar. **B.** Box-Whisker plots of *ADORA2B* methylation level in thyroid tumors with or without mutations in PI3K pathway. The mutations in the PI3K pathway included any mutations in *PIK3CA*, *PTEN*, and the three *RAS* genes. The box-plot shows the five statistics (lower whisker is 5% minimum, lower box part is the 25th percentile, solid line in box presents the median, upper box part is 75th percentile, and upper whisker is 95% maximum).

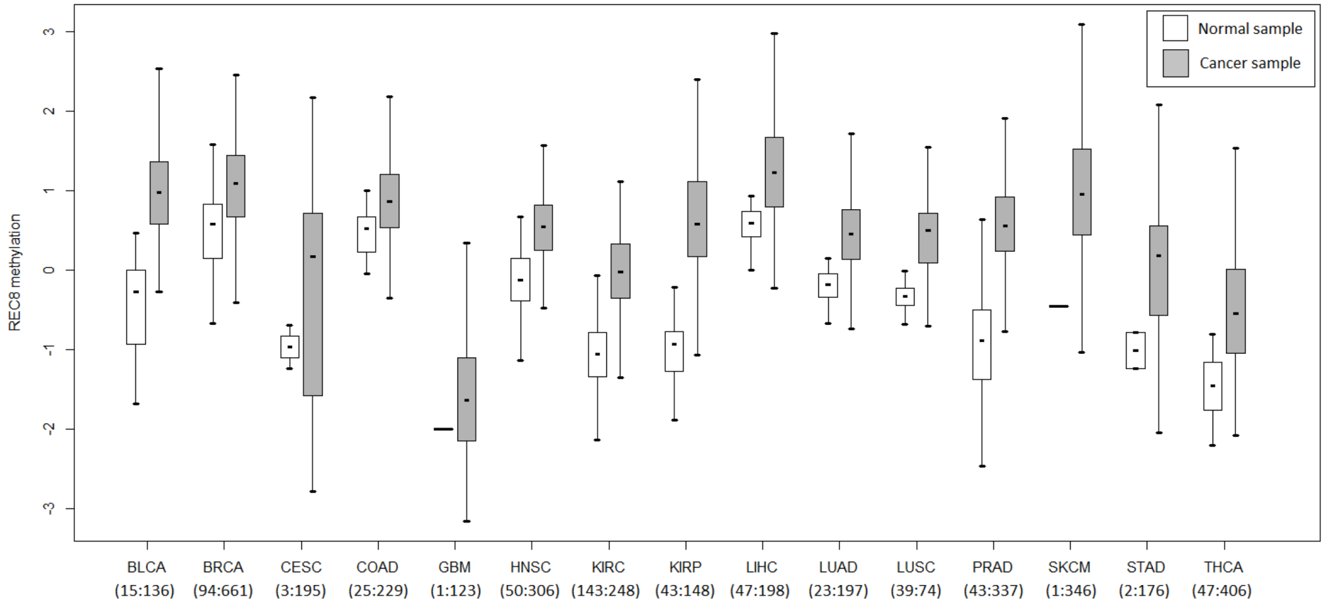

Supplementary Figure S4: *REC8* hypermethylation in various human cancers in the TCGA database. Shown are 15 types of cancer in the TCGA database, in which we observed that the methylation level of REC8 in cancer samples (gray color) was consistently higher than that in the corresponding normal samples (white color). The box-plot shows the five statistics (the lower whisker is 5% minimum, the lower box part is the 25th percentile, the solid line in box presents the median, the upper box part is 75th percentile, and the upper whisker is 95% maximum). Symbols BLCA, BRCA, CESC, COAD, GBM, HNSC, KIRC, KIRP, LIHC, LUAD, LUSC, PRAD, SKCM, STAD, and THCA are the abbreviations for ladder urothelial carcinoma, breast invasive carcinoma, cervical squamous cell carcinoma and endocervical adenocarcinoma, colon adenocarcinoma, glioblastoma multiforme, head and neck squamous cell carcinoma, kidney renal clear cell carcinoma, kidney renal papillary cell carcinoma, liver hepatocellular carcinoma, lung adenocarcinoma, lung squamous cell carcinoma, prostate adenocarcinoma, skin cutaneous melanoma, stomach adenocarcinoma, and thyroid carcinoma, respectively. The numbers in the brackets under each cancer type represent the numbers of normal samples:the tumor samples for each cancer type.

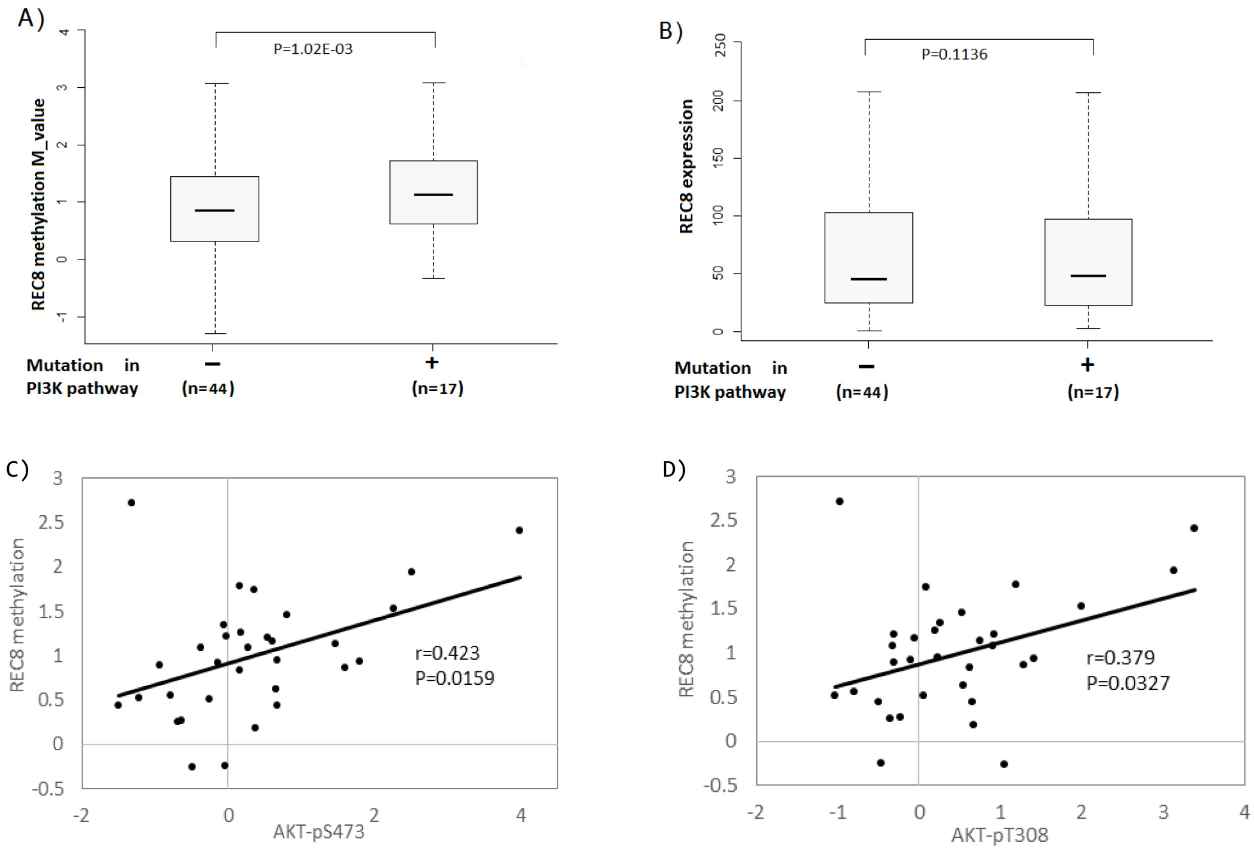

Supplementary Figure S5: Relationship between *REC8* methylation/expression and genetic alterations/signaling activities of the PI3K pathway in primary cutaneous melanoma in the TCGA database. A. Association between *REC8* hypermethylation and genetic alterations in the PI3K pathway in primary melanoma. The genetic alterations included mutations in *PIK3CA*, *PTEN* and the three *RAS* genes. **B**. Inverse association between the REC8 mRNA expression and the above genetic alterations in the PI3K pathway **C**. Association between REC8 hypermethylation and AKT-pS473 phosphorylation in primary melanoma samples. **D**. Association between *REC8* hypermethylation and AKT- pT3083 phosphorylation in primary melanoma samples.

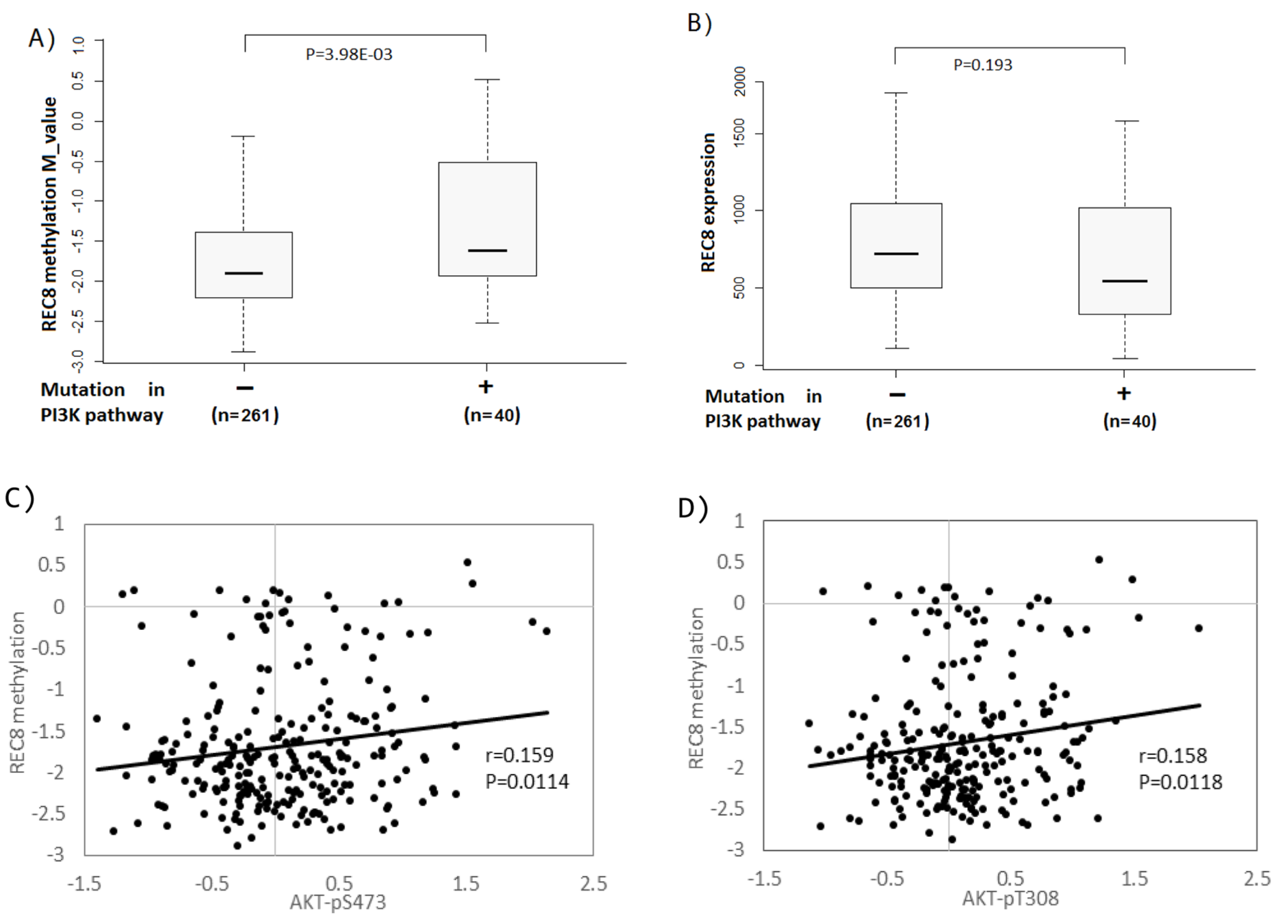

Supplementary Figure S6: Relationship between *REC8* methylation/expression and genetic alterations/signaling activities of the PI3K pathway in low-grade glioma in the TCGA database. A. Association between *REC8* hypermethylation and genetic alterations in PI3K pathway in glioma. The genetic alterations included mutations in *PIK3CA*, *PTEN* and the three *RAS* genes. **B.** Trend of inverse association between the *REC8* mRNA expression and the above genetic alterations in the PI3K pathway. **C.** Association between *REC8* hypermethylation and AKT-pS473 phosphorylation. **D.** Association between *REC8* hypermethylation and AKT-pT308 phosphorylation.

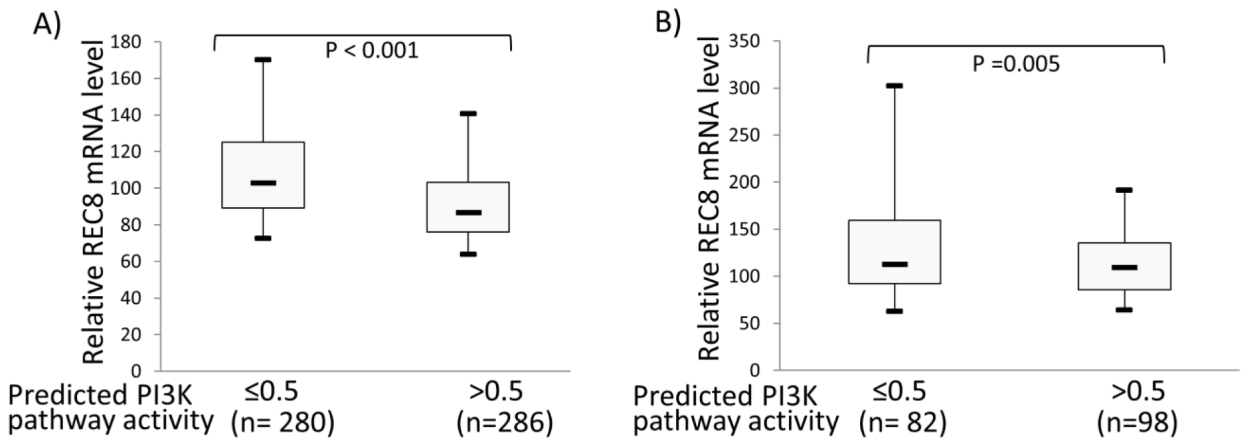

Supplementary Figure S7: Relationship between *REC8* expression and the PI3K pathway activity in colon cancer and melanoma in the database probeset: 218599. The expression data of *REC8* (probeset: 218599_at) in tumor samples were retrieved from the RMA-normalized microarray dataset. The predicted PI3K pathway activities (ranging from 0 to 1) of each tumor sample were calculated by BinReg algorithm as described in Supplementary Materials and Methods. The box-plot shows the five statistics (5th, 25th, 50th, 75th and 95th percentile) as described in the legend to Figure 5). Randomization test was used to calculate the *p*-values for the difference of expression level in the two tumor groups. **A.** Box-Whisker plots of *REC8* expression level in colon cancer, which was significantly higher in the group with lower PI3K pathway activities (≤0.5) than in the group with higher PI3K pathway activities (>0.5). **B**. Box-Whisker plots of *REC8* expression level in melanoma, which was significantly higher in the group with lower PI3K pathway activities (≤0.5) than in the group with higher PI3K pathway activities (>0.5).

Supplementary Table S1: Genes significantly hypomethylated or hypermethylated after MK2006 treatment in thyroid cancer cell lines.

Supplementary Table S2: Gene genetic/epigenetic analysis results for the thyroid samples using in this study.

Supplementary Table S3: REC8 mRNA level and predicted PI3K pathway activity for individual colon cancer or melanoma samples.

Supplementary Table S4: Primers used in this study

Gene symbol	Forward (5′-3′)	Reverse (5′-3′)	Size (bp)
MSP primer			
CCNA1	TTTCGAGGATTTCGCGTCGT	CTCCTAAAAACCCTAACTCGA	47
REC8	GAATTTTGTGTTTTAAAGAATTTC	ATAACAAAACAACCGATATAACG	97
GSPT1	ATTTGTAAGTGTAAGATGTAGTC	ACCGTATTCCATAATATATCCG	158
PSMD6	CGGAGACGGGATCGGAAGTC	TTAATTACGACCGACTACGACAACG	135
CDKN2D	TTTGTCGGGTTTATTTGAAAATC	AAAATATAACAACCGATAAACCG	63
TCF7L2	AAGGATTATTGTTAGTCGTTTTC	CACACTACCTTAATAAAATACCG	102
ATG4C	ATGTTGGTTAGGTTGGTTTCG	AACACAAAACAATCGAACGCG	108
ADORA2B	ATTGTTACGGGTTGGCGAAC	ACACCCTCAAATCTACATACG	99
RPTOR	ATTAAAATTAAGGAGTATTCGCG	TACATACAACGAACGCTTACG	61
RT-PCR primer			
REC8	TTGGTGAAGCGCGAATACCTG	CTCGTACCAGCACGTAATTGAG	76
GSPT1	TGGACGCACATTTGATGCC	CCACCTCCTCAATACAGGTATGA	104
CDKN2D	CCTCAACCGCTTCGGCAAGA	GACATTGGGGCTGGCACCTT	96
ADORA2B	TGCACTGACTTCTACGGCTG	GGTCCCCGTGACCAAACTT	147
