# Supplementary material for: REC8 is a novel tumor suppressor gene epigenetically robustly targeted by the PI3K pathway in thyroid cancer
Source: Oncotarget. 2015 Oct 13;6(36):39211–24. doi: 10.18632/oncotarget.5391 (PMC4770767; doi:10.18632/oncotarget.5391)
Supplement: Supplementary file 2 [file oncotarget-06-39211-s002.docx]

**Supplementary Table 1. Genes significantly hypomethylated or hypermethylated after MK2006treatment in thyroid cancer cell lines**

| Probe | Gene | Location | Enhancer | Is_CpG  _Island | Chromo_  some | Map_infomation | Fold change (MK2006/DMSO)  FTC133 K1 OCUT1 | | | p-value |
| --- | --- | --- | --- | --- | --- | --- | --- | --- | --- | --- |
| Methylation increased by MK2006 | | | | | | | | | | |
| cg05059616 | RASA3 | 3'UTR | TRUE | 0 | 13 | 114747479 | 3.28 | 2.44 | 2.7 | 4.10E-06 |
| cg02078039 | ZNRD1 | TSS1500 |  | 0 | 6 | 30028257 | 2.03 | 2.6 | 1.99 | 4.04E-05 |
| cg22838399 | EFNA5 | Body | TRUE | 0 | 5 | 106912785 | 2.11 | 2.6 | 1.81 | 6.29E-05 |
| cg24246628 | KIF25 | Body | TRUE | 1 | 6 | 168435914 | 2.02 | 1.84 | 2.58 | 6.33E-05 |
| cg12181579 | RBM19 | Body | TRUE | 1 | 12 | 114312919 | 2.16 | 1.72 | 2.59 | 8.81E-05 |
| cg04566778 | RANBP9 | TSS200 |  | 1 | 6 | 13711921 | 1.92 | 2.19 | 1.84 | 9.19E-05 |
| cg11717782 | PPFIA4 | Body | TRUE | 0 | 1 | 203023927 | 1.93 | 1.88 | 1.99 | 9.93E-05 |
| cg25830379 | IGF1R | Body | TRUE | 0 | 15 | 99264361 | 2.5 | 1.62 | 2.63 | 1.05E-04 |
| cg24061105 | ATP9B | Body | TRUE | 1 | 18 | 77125858 | 1.93 | 1.82 | 2.06 | 1.05E-04 |
| cg21470600 | NPHP4 | Body | TRUE | 0 | 1 | 6040824 | 1.75 | 2.33 | 1.99 | 1.06E-04 |
| cg15834072 | DCHS2 | 1stExon |  | 1 | 4 | 155411603 | 2.06 | 1.98 | 1.8 | 1.10E-04 |
| cg19593550 | SNORD41 | TSS200 |  | 0 | 19 | 12817426 | 1.73 | 2.15 | 2.1 | 1.12E-04 |
| cg25083618 | CYP26C1 | TSS200 |  | 1 | 10 | 94820988 | 1.71 | 2.32 | 2.05 | 1.16E-04 |
| cg18247055 | SPAG6 | TSS200 |  | 1 | 10 | 22634226 | 2.03 | 2.22 | 1.71 | 1.20E-04 |
| cg20667480 | TBC1D16 | Body | TRUE | 1 | 17 | 77916768 | 1.72 | 2.03 | 2.14 | 1.23E-04 |
| cg03148084 | CNDP1 | TSS200 |  | 0 | 18 | 72201665 | 1.91 | 2.71 | 1.76 | 1.25E-04 |
| cg00307254 | PLXNA1 | TSS1500 |  | 0 | 3 | 126706825 | 1.98 | 1.86 | 3.73 | 1.36E-04 |
| cg15003763 | TMEFF2 | Body | TRUE | 0 | 2 | 192950079 | 2.15 | 1.7 | 1.97 | 1.46E-04 |
| cg07637837 | MBP | 5'UTR |  | 1 | 18 | 74824154 | 1.7 | 1.93 | 2.63 | 1.49E-04 |
| cg14695497 | GALNT9 | Body | TRUE | 1 | 12 | 132860966 | 2.12 | 1.7 | 1.99 | 1.55E-04 |
| cg09933215 | SDC3 | Body | TRUE | 0 | 1 | 31360239 | 1.9 | 1.7 | 2.16 | 1.60E-04 |
| cg09015921 | RAI1 | 3'UTR | TRUE | 1 | 17 | 17714347 | 1.93 | 2.01 | 1.73 | 1.61E-04 |
| cg13792579 | GPSM1 | Body | TRUE | 1 | 9 | 139237366 | 2.04 | 1.74 | 1.88 | 1.62E-04 |
| cg06235582 | HBA1 | TSS200 |  | 1 | 16 | 226668 | 2 | 1.71 | 1.92 | 1.66E-04 |
| cg27661460 | PIEZO1 | Body | TRUE | 1 | 16 | 88844969 | 2.54 | 1.57 | 2.29 | 1.71E-04 |
| cg03644281 | LOC221442 | TSS200 | TRUE | 1 | 6 | 41068752 | 2.2 | 2.95 | 1.58 | 1.72E-04 |
| cg26301689 | PAQR9 | 1stExon |  | 1 | 3 | 142681704 | 1.72 | 1.84 | 2.43 | 1.74E-04 |
| cg02478448 | CCNA1 | 1stExon;5'UTR;TSS1500 | TRUE | 1 | 13 | 37006063 | 1.77 | 2.23 | 1.78 | 1.78E-04 |
| cg22884541 | BHLHA15 | 1stExon |  | 1 | 7 | 97841864 | 1.95 | 1.87 | 1.78 | 1.78E-04 |
| cg16937327 | MAGEA8 | 5'UTR;1stExon;1stExon |  | 0 |  | 149010059 | 2.12 | 1.7 | 1.84 | 1.85E-04 |
| cg05729372 | PRKG1 | Body | TRUE | 0 | 10 | 53526990 | 1.87 | 1.68 | 2.17 | 1.86E-04 |
| cg14237746 | HOXB8 | TSS1500 |  | 1 | 17 | 46692592 | 1.77 | 1.9 | 1.84 | 1.93E-04 |
| cg21644334 | AMPD2 | Body;5'UTR;1stExon | TRUE | 0 | 1 | 110168049 | 1.61 | 2.22 | 3.63 | 2.10E-04 |
| cg17367832 | MIR149 | TSS200 |  | 1 | 2 | 241395288 | 1.62 | 2.16 | 1.92 | 2.26E-04 |
| cg22857947 | PRKACA | TSS200;Body |  | 0 | 19 | 14225039 | 1.94 | 2 | 1.61 | 2.36E-04 |
| cg06188670 | DKK3 | TSS200;TSS1500;5'UTR |  | 1 | 11 | 12030639 | 1.56 | 1.98 | 2.22 | 2.37E-04 |
| cg24580076 | GET4 | TSS1500 |  | 1 | 7 | 915073 | 1.74 | 2.94 | 1.77 | 2.49E-04 |
| cg11703729 | FRMD4A | Body | TRUE | 0 | 10 | 13851615 | 1.83 | 1.62 | 2.35 | 2.50E-04 |
| cg23541019 | C14orf79 | TSS1500 |  | 1 | 14 | 105451561 | 1.89 | 1.6 | 2.52 | 2.50E-04 |
| cg21270847 | PBX1 | Body | TRUE | 1 | 1 | 164604806 | 1.79 | 1.69 | 1.98 | 2.50E-04 |
| cg01511465 | MC1R | 1stExon | TRUE | 1 | 16 | 89986185 | 1.54 | 1.97 | 2.28 | 2.75E-04 |
| cg27143246 | MYNN | TSS1500 |  | 0 | 3 | 169489583 | 2.02 | 1.58 | 1.94 | 2.75E-04 |
| cg23912877 | SLC22A18AS | Body;TSS1500 |  | 0 | 11 | 2926337 | 1.62 | 2.06 | 1.86 | 2.77E-04 |
| cg11686940 | KLF13 | Body | TRUE | 1 | 15 | 31664433 | 1.52 | 2.02 | 2.41 | 2.81E-04 |
| cg08539872 | UNC5B | Body | TRUE | 1 | 10 | 73004610 | 1.59 | 2.76 | 1.9 | 2.89E-04 |
| cg21887544 | TRAPPC10 | TSS1500 |  | 0 | 21 | 45431138 | 1.62 | 1.94 | 1.85 | 3.01E-04 |
| cg03104758 | FAM192A | 1stExon |  | 0 | 16 | 57219801 | 2.04 | 1.56 | 1.93 | 3.04E-04 |
| cg03104758 | RSPRY1 | TSS1500 |  | 0 | 16 | 57219801 | 2.04 | 1.56 | 1.93 | 3.04E-04 |
| cg06976003 | GLYR1 | 1stExon |  | 1 | 16 | 4897242 | 2.06 | 1.77 | 1.91 | 3.04E-04 |
| cg06976003 | UBN1 | TSS1500 |  | 1 | 16 | 4897242 | 2.06 | 1.77 | 1.91 | 3.04E-04 |
| cg04128884 | PCGF3 | 5'UTR | TRUE | 1 | 4 | 714516 | 1.9 | 2.9 | 1.57 | 3.07E-04 |
| cg01211124 | NUPL1 | TSS1500 |  | 0 | 13 | 25874859 | 1.73 | 1.64 | 2.25 | 3.14E-04 |
| cg27206976 | PLXNB2 | 5'UTR | TRUE | 1 | 22 | 50738315 | 1.75 | 1.75 | 1.82 | 3.21E-04 |
| cg19626656 | C1orf159 | TSS1500 |  | 1 | 1 | 1053027 | 1.52 | 1.96 | 2.72 | 3.37E-04 |
| cg03985192 | HDAC4 | 5'UTR |  | 1 | 2 | 240288716 | 1.66 | 1.74 | 1.84 | 3.75E-04 |
| cg05233946 | PRR14L | 3'UTR | TRUE | 0 | 22 | 32078088 | 2.06 | 1.51 | 1.91 | 3.88E-04 |
| cg15208757 | IGFBP3 | TSS1500 |  | 1 | 7 | 45961943 | 1.63 | 1.75 | 1.86 | 3.96E-04 |
| cg22618164 | WDR66 | TSS200 | TRUE | 1 | 12 | 122356400 | 1.6 | 1.98 | 1.76 | 4.01E-04 |
| cg20749790 | SLC23A2 | 5'UTR;TSS200 |  | 1 | 20 | 4982291 | 1.82 | 1.67 | 1.72 | 4.35E-04 |
| cg06938264 | PCDHA7 | 1stExon |  | 1 | 5 | 140214372 | 1.85 | 1.76 | 1.61 | 4.45E-04 |
| cg00632861 | ZC3H13 | 5'UTR |  | 1 | 13 | 46626485 | 1.58 | 1.7 | 2.47 | 4.49E-04 |
| cg21038220 | PCDHB7 | TSS1500 |  | 0 | 5 | 140551968 | 1.61 | 1.84 | 3.54 | 4.70E-04 |
| cg20462129 | RPTOR | Body | TRUE | 1 | 17 | 78607095 | 1.62 | 1.66 | 2.06 | 4.70E-04 |
| cg03588299 | DIP2C | Body | TRUE | 1 | 10 | 390961 | 1.87 | 1.51 | 1.92 | 4.77E-04 |
| cg08994763 | C3orf21 | Body | TRUE | 0 | 3 | 194822510 | 2.19 | 1.69 | 1.57 | 4.80E-04 |
| cg07926092 | RNF219 | TSS200 |  | 0 | 13 | 79233506 | 1.81 | 1.62 | 1.73 | 4.95E-04 |
| cg16480692 | ZNF75A | TSS200 |  | 1 | 16 | 3355295 | 1.76 | 1.8 | 1.6 | 5.02E-04 |
| cg04128092 | CAMTA1 | Body | TRUE | 0 | 1 | 7747160 | 1.61 | 2.65 | 1.66 | 5.17E-04 |
| cg02968175 | MGRN1 | Body | TRUE | 1 | 16 | 4690020 | 1.63 | 2.78 | 1.64 | 5.17E-04 |
| cg04731448 | SCT | TSS1500 | TRUE | 1 | 11 | 628628 | 1.53 | 1.89 | 1.83 | 5.19E-04 |
| cg13751417 | VAV2 | Body | TRUE | 0 | 9 | 136814406 | 1.53 | 1.77 | 1.94 | 5.24E-04 |
| cg14696311 | RASA3 | Body | TRUE | 1 | 13 | 114855198 | 2.03 | 1.73 | 1.54 | 5.25E-04 |
| cg04253037 | DDX56 | TSS1500 |  | 0 | 7 | 44614489 | 1.54 | 2.58 | 1.73 | 5.27E-04 |
| cg16889990 | USP29 | TSS200 |  | 0 | 19 | 57631478 | 1.61 | 1.91 | 1.66 | 5.34E-04 |
| cg02100381 | COL4A2 | Body | TRUE | 0 | 13 | 111025711 | 1.63 | 1.8 | 1.69 | 5.51E-04 |
| cg11245569 | TRIM66 | 5'UTR | TRUE | 0 | 11 | 8679885 | 1.66 | 2.39 | 1.54 | 6.04E-04 |
| cg04665351 | TULP3 | TSS200 |  | 1 | 12 | 3000000 | 2.13 | 1.58 | 1.62 | 6.12E-04 |
| cg22268467 | SH3GL1 | Body | TRUE | 1 | 19 | 4361713 | 1.85 | 1.55 | 1.68 | 6.61E-04 |
| cg06029846 | PRMT8 | TSS200 |  | 1 | 12 | 3600317 | 1.91 | 1.57 | 1.63 | 6.80E-04 |
| cg14627760 | NPB | Body | TRUE | 1 | 17 | 79860435 | 1.76 | 1.64 | 1.63 | 6.85E-04 |
| cg00384577 | ADCK4 | 1stExon |  | 1 | 19 | 41223078 | 1.55 | 1.74 | 1.7 | 6.88E-04 |
| cg00384577 | ITPKC | TSS1500 |  | 1 | 19 | 41223078 | 1.55 | 1.74 | 1.7 | 6.88E-04 |
| cg08549326 | ST7 | Body | TRUE | 0 | 7 | 116617896 | 1.7 | 1.51 | 1.91 | 7.05E-04 |
| cg15168615 | DDX54 | Body | TRUE | 1 | 12 | 113599683 | 1.66 | 1.73 | 1.62 | 7.07E-04 |
| cg07930552 | C6orf192 | 5'UTR;1stExon |  | 1 | 6 | 133119739 | 1.51 | 1.65 | 2.23 | 7.30E-04 |
| cg26562141 | CTBP2 | 5'UTR | TRUE | 0 | 10 | 126805095 | 1.7 | 1.62 | 1.67 | 7.40E-04 |
| cg22421148 | BLCAP | TSS1500;5'UTR |  | 1 | 20 | 36149119 | 1.6 | 1.61 | 1.79 | 7.74E-04 |
| cg22421148 | NNAT | TSS1500;5'UTR |  | 1 | 20 | 36149119 | 1.6 | 1.61 | 1.79 | 7.74E-04 |
| cg22222092 | BIVM | TSS1500 |  | 0 | 13 | 103450385 | 1.57 | 1.65 | 1.77 | 7.79E-04 |
| cg00945932 | EXTL3 | 5'UTR |  | 1 | 8 | 28559273 | 1.53 | 1.6 | 2.12 | 7.95E-04 |
| cg14834267 | NAV1 | 1stExon |  | 1 | 1 | 201617847 | 1.64 | 1.79 | 1.57 | 8.04E-04 |
| cg27308021 | C16orf73 | TSS200 |  | 1 | 16 | 1922207 | 1.74 | 1.7 | 1.53 | 8.55E-04 |
| cg26287152 | TBC1D16 | Body | TRUE | 1 | 17 | 77923675 | 1.58 | 1.65 | 1.72 | 8.56E-04 |
| cg03529432 | HOXA6 | TSS200 |  | 1 | 7 | 27187502 | 1.59 | 1.54 | 2 | 8.68E-04 |
| cg21041100 | RAB5B | TSS200 |  | 1 | 12 | 56367818 | 1.53 | 2.45 | 1.55 | 8.75E-04 |
| cg03698372 | DTX2 | Body;1stExon |  | 0 | 7 | 76110038 | 1.54 | 1.67 | 1.73 | 8.91E-04 |
| cg22901337 | PABPN1 | 1stExon;5'UTR |  | 1 | 14 | 23790498 | 1.75 | 1.71 | 1.52 | 9.03E-04 |
| cg18661731 | KIAA1468 | TSS1500 |  | 1 | 18 | 59854239 | 1.66 | 1.55 | 1.75 | 9.09E-04 |
| cg18661731 | PIGN | 5'UTR;1stExon |  | 1 | 18 | 59854239 | 1.66 | 1.55 | 1.75 | 9.09E-04 |
| cg12712747 | COL4A1 | Body | TRUE | 1 | 13 | 110900663 | 1.69 | 1.64 | 1.57 | 9.35E-04 |
| cg03698359 | TRIM5 | 5'UTR |  | 1 | 11 | 5704417 | 1.82 | 1.55 | 1.6 | 9.62E-04 |
| cg00965330 | ZNF182 | Body;5'UTR |  | 0 |  | 47861943 | 1.57 | 1.99 | 1.52 | 9.83E-04 |
| cg15827779 | CDC42BPB | TSS1500 |  | 1 | 14 | 103524899 | 1.57 | 1.71 | 1.58 | 1.03E-03 |
| cg17749509 | SLC16A4 | TSS1500 | TRUE | 0 | 1 | 110934279 | 1.5 | 1.79 | 1.64 | 1.05E-03 |
| cg23323712 | MACROD1 | Body | TRUE | 1 | 11 | 63851382 | 1.7 | 1.58 | 1.59 | 1.05E-03 |
| cg18279337 | MYO5C | TSS200 |  | 1 | 15 | 52588166 | 1.69 | 1.67 | 1.53 | 1.06E-03 |
| cg14125368 | DDX31 | Body | TRUE | 0 | 9 | 135474365 | 1.59 | 1.78 | 1.53 | 1.10E-03 |
| cg09647671 | SPATA2 | 5'UTR;TSS200 |  | 0 | 20 | 48530454 | 1.68 | 1.65 | 1.53 | 1.10E-03 |
| cg20313298 | PCGF3 | 5'UTR |  | 1 | 4 | 708468 | 1.76 | 1.52 | 1.61 | 1.15E-03 |
| cg18649601 | ZC3H11A | TSS200 |  | 1 | 1 | 203764618 | 1.83 | 1.63 | 1.52 | 1.15E-03 |
| cg06813419 | TRAF5 | 5'UTR | TRUE | 0 | 1 | 211502171 | 1.57 | 1.65 | 1.6 | 1.21E-03 |
| cg22254463 | EXPH5 | 5'UTR;Body | TRUE | 0 | 11 | 108422663 | 1.52 | 1.55 | 1.79 | 1.25E-03 |
| cg17202331 | BTBD3 | 5'UTR;TSS1500 |  | 0 | 20 | 11897453 | 1.69 | 1.59 | 1.52 | 1.30E-03 |
| cg26001902 | SLC5A7 | TSS200 |  | 1 | 2 | 108602979 | 1.75 | 1.54 | 1.54 | 1.31E-03 |
| cg13259205 | DKK3 | TSS200;5'UTR |  | 1 | 11 | 12030375 | 1.53 | 1.52 | 2.93 | 1.33E-03 |
| cg17335114 | MTDH | TSS200 |  | 1 | 8 | 98656329 | 1.75 | 1.56 | 1.5 | 1.35E-03 |
| cg09756865 | ATG4C | TSS1500 |  | 0 | 1 | 63249197 | 1.56 | 1.78 | 1.52 | 1.38E-03 |
| cg24786549 | LSM6 | TSS200 |  | 0 | 4 | 147096785 | 1.54 | 1.7 | 1.54 | 1.40E-03 |
| cg14614094 | EXOSC2 | TSS1500 |  | 1 | 9 | 133567903 | 1.64 | 1.52 | 1.6 | 1.50E-03 |
| cg21158411 | PIP5KL1 | Body;5'UTR;1stExon |  | 1 | 9 | 130689627 | 1.56 | 1.72 | 1.51 | 1.51E-03 |
| cg24289669 | CUL4A | 5'UTR;Body |  | 0 | 13 | 113866760 | 1.75 | 1.5 | 1.52 | 1.60E-03 |
| cg08826152 | ADORA2B | Body | TRUE | 0 | 17 | 15869607 | 1.54 | 1.56 | 1.6 | 1.79E-03 |
| cg17994139 | HOXA6 | TSS200 |  | 1 | 7 | 27187556 | 1.65 | 1.52 | 1.53 | 1.89E-03 |
| cg05571558 | MIR885 | TSS200 | TRUE | 0 | 3 | 10436304 | 1.55 | 1.52 | 1.54 | 2.37E-03 |
| cg26502583 | KLHL10 | TSS1500 |  | 1 | 17 | 39992600 | 1.51 | 1.56 | 1.51 | 2.43E-03 |
| cg26502583 | NT5C3L | TSS200 |  | 1 | 17 | 39992600 | 1.51 | 1.56 | 1.51 | 2.43E-03 |
| cg12550574 | SPCS3 | TSS1500 |  | 0 | 4 | 177240810 | 1.52 | 2.31 | 2.15 | 2.44E-03 |
| cg04599342 | ZNF541 | 1stExon |  | 0 | 19 | 48058991 | 1.53 | 1.53 | 1.53 | 2.49E-03 |
| cg10789050 | OSBPL10 | Body | TRUE | 0 | 3 | 31713847 | 1.53 | 1.53 | 1.51 | 2.53E-03 |
| cg09289469 | PPA2 | Body | TRUE | 0 | 4 | 106321260 | 1.57 | 1.51 | 1.51 | 2.60E-03 |
| cg01064898 | MTMR6 | 1stExon;5'UTR |  | 1 | 13 | 25861655 | 1.5 | 1.55 | 1.5 | 2.73E-03 |

methylation decreased by MK2006

| cg22898729 | C6orf145 | Body | TRUE | 1 | 6 | 3742668 | 0.56 | 0.42 | 0.36 | 4.54E-05 |
| --- | --- | --- | --- | --- | --- | --- | --- | --- | --- | --- |
| cg24770230 | AP3S1 | Body | TRUE | 0 | 5 | 115194332 | 0.49 | 0.4 | 0.55 | 7.26E-05 |
| cg23874746 | PDE1A | 1stExon;Body |  | 0 | 2 | 183387015 | 0.51 | 0.53 | 0.51 | 1.04E-04 |
| cg24785316 | ZNF827 | Body | TRUE | 0 | 4 | 146815316 | 0.39 | 0.45 | 0.63 | 1.42E-04 |
| cg22450146 | MFSD11 | TSS200 |  | 1 | 17 | 74733660 | 0.63 | 0.37 | 0.26 | 1.57E-04 |
| cg22450146 | MIR636 | TSS1500 |  | 1 | 17 | 74733660 | 0.63 | 0.37 | 0.26 | 1.57E-04 |
| cg22450146 | SFRS2 | TSS1500 |  | 1 | 17 | 74733660 | 0.63 | 0.37 | 0.26 | 1.57E-04 |
| cg16273152 | RBM44 | 5'UTR |  | 1 | 2 | 238708018 | 0.59 | 0.52 | 0.38 | 1.62E-04 |
| cg22655038 | SMTN | TSS200 |  | 1 | 22 | 31477112 | 0.59 | 0.52 | 0.5 | 1.71E-04 |
| cg12462078 | PDE11A | TSS1500;Body |  | 0 | 2 | 178788607 | 0.55 | 0.57 | 0.48 | 1.77E-04 |
| cg14694234 | TCEAL4 | TSS1500 |  | 0 |  | 102839366 | 0.55 | 0.41 | 0.59 | 1.80E-04 |
| cg15579587 | LRRFIP1 | TSS1500;Body |  | 1 | 2 | 238600061 | 0.51 | 0.59 | 0.51 | 1.92E-04 |
| cg25613170 | TBKBP1 | TSS200 |  | 1 | 17 | 45772523 | 0.53 | 0.45 | 0.6 | 1.98E-04 |
| cg07279281 | ALS2CL | TSS200 |  | 1 | 3 | 46735319 | 0.61 | 0.54 | 0.44 | 2.01E-04 |
| cg09805403 | GSPT1 | TSS1500;TSS200 |  | 0 | 16 | 12010698 | 0.54 | 0.57 | 0.53 | 2.23E-04 |
| cg20495040 | SLC50A1 | TSS1500 |  | 1 | 1 | 155107961 | 0.58 | 0.59 | 0.47 | 2.56E-04 |
| cg03065625 | LRP4 | TSS1500 | TRUE | 0 | 11 | 46941490 | 0.63 | 0.53 | 0.47 | 2.57E-04 |
| cg20064151 | TXNIP | 1stExon |  | 1 | 1 | 145438865 | 0.67 | 0.47 | 0.47 | 2.65E-04 |
| cg10062617 | TSSC4 | TSS1500 |  | 0 | 11 | 2422632 | 0.5 | 0.39 | 0.65 | 2.67E-04 |
| cg05338317 | CDKN2D | TSS1500 |  | 1 | 19 | 10679934 | 0.51 | 0.63 | 0.54 | 2.82E-04 |
| cg08101918 | NLGN4X | 5'UTR;TSS1500;1stExon |  | 1 |  | 6146656 | 0.55 | 0.62 | 0.54 | 3.35E-04 |
| cg06181463 | PSMD6 | TSS200 |  | 1 | 3 | 64009142 | 0.53 | 0.27 | 0.6 | 3.38E-04 |
| cg18730034 | GABRB3 | Body | TRUE | 0 | 15 | 26866630 | 0.66 | 0.51 | 0.39 | 3.47E-04 |
| cg23416909 | USP6 | TSS200 |  | 0 | 17 | 5031675 | 0.66 | 0.52 | 0.49 | 3.77E-04 |
| cg01188822 | POLR1C | TSS200 |  | 1 | 6 | 43484785 | 0.6 | 0.59 | 0.53 | 3.86E-04 |
| cg01188822 | YIPF3 | TSS200 |  | 1 | 6 | 43484785 | 0.6 | 0.59 | 0.53 | 3.86E-04 |
| cg15768138 | CXCR1 | 5'UTR |  | 0 | 2 | 219030752 | 0.56 | 0.56 | 0.6 | 3.89E-04 |
| cg20118431 | B3GAT1 | 5'UTR |  | 0 | 11 | 134278896 | 0.63 | 0.61 | 0.48 | 5.39E-04 |
| cg20721738 | RAB36 | TSS1500 |  | 0 | 22 | 23487104 | 0.61 | 0.41 | 0.64 | 5.83E-04 |
| cg18363192 | NEU1 | TSS1500 |  | 1 | 6 | 31831434 | 0.6 | 0.58 | 0.61 | 6.38E-04 |
| cg24329557 | GCM2 | TSS1500 |  | 1 | 6 | 10882326 | 0.61 | 0.6 | 0.58 | 6.79E-04 |
| cg08723769 | RPL13 | TSS200 |  | 1 | 16 | 89627049 | 0.58 | 0.63 | 0.58 | 6.91E-04 |
| cg08723769 | SNORD68 | TSS1500 |  | 1 | 16 | 89627049 | 0.58 | 0.63 | 0.58 | 6.91E-04 |
| cg07639483 | ZBED4 | TSS1500 |  | 1 | 22 | 50247176 | 0.66 | 0.52 | 0.61 | 7.26E-04 |
| cg01405821 | TTL | Body | TRUE | 0 | 2 | 113250679 | 0.61 | 0.61 | 0.59 | 7.49E-04 |
| cg03437886 | RALGPS2 | 5'UTR |  | 0 | 1 | 178698946 | 0.5 | 0.64 | 0.63 | 7.64E-04 |
| cg16360659 | GPX6 | TSS1500 |  | 0 | 6 | 28484320 | 0.62 | 0.49 | 0.65 | 7.74E-04 |
| cg12896146 | MTNR1A | TSS1500 |  | 0 | 4 | 187477065 | 0.65 | 0.46 | 0.63 | 7.92E-04 |
| cg20129213 | RIMS2 | TSS1500 |  | 1 | 8 | 104512317 | 0.62 | 0.49 | 0.66 | 8.00E-04 |
| cg15043711 | HSPA1B | 3'UTR;1stExon |  | 0 | 6 | 31797954 | 0.6 | 0.56 | 0.65 | 8.29E-04 |
| cg20445883 | CYBASC3 | TSS200;Body |  | 1 | 11 | 61129875 | 0.64 | 0.67 | 0.42 | 8.46E-04 |
| cg20445883 | TMEM138 | 1stExon;5'UTR |  | 1 | 11 | 61129875 | 0.64 | 0.67 | 0.42 | 8.46E-04 |
| cg12600418 | ZC3H12A | TSS200 |  | 1 | 1 | 37939923 | 0.62 | 0.65 | 0.59 | 9.82E-04 |
| cg14737332 | TCF7L2 | TSS200 |  | 1 | 10 | 114709880 | 0.66 | 0.56 | 0.62 | 9.82E-04 |
| cg11834844 | ASNA1 | TSS200 |  | 1 | 19 | 12848279 | 0.63 | 0.64 | 0.58 | 1.02E-03 |
| cg00184016 | ATXN2L | TSS1500 |  | 0 | 16 | 28833823 | 0.65 | 0.66 | 0.39 | 1.06E-03 |
| cg05405389 | DLG2 | Body | TRUE | 0 | 11 | 84386472 | 0.64 | 0.65 | 0.54 | 1.06E-03 |
| cg16485682 | GATA6 | 5'UTR;1stExon |  | 1 | 18 | 19749540 | 0.66 | 0.35 | 0.65 | 1.19E-03 |
| cg10158541 | CCNA1 | 1stExon;5'UTR;TSS200 |  | 1 | 13 | 37006265 | 0.66 | 0.66 | 0.53 | 1.26E-03 |
| cg04915494 | FLYWCH1 | 5'UTR |  | 0 | 16 | 2962968 | 0.66 | 0.38 | 0.67 | 1.35E-03 |
| cg08843517 | CYBA | TSS200 |  | 1 | 16 | 88717464 | 0.61 | 0.66 | 0.61 | 1.47E-03 |
| cg11026604 | GABRB3 | TSS1500 |  | 0 | 15 | 27020039 | 0.6 | 0.66 | 0.64 | 1.52E-03 |
| cg25667409 | SLC7A2 | Body;5'UTR |  | 0 | 8 | 17399899 | 0.63 | 0.64 | 0.64 | 1.55E-03 |
| cg18512948 | REC8 | 1stExon;5'UTR | TRUE | 1 | 14 | 24641706 | 0.61 | 0.63 | 0.66 | 1.59E-03 |
| cg03424058 | KCTD3 | Body | TRUE | 0 | 1 | 215791864 | 0.66 | 0.63 | 0.62 | 1.68E-03 |
| cg04308657 | FMNL2 | Body | TRUE | 0 | 2 | 153362242 | 0.63 | 0.66 | 0.65 | 1.82E-03 |
| cg12900942 | THRAP3 | TSS1500 |  | 1 | 1 | 36689399 | 0.65 | 0.63 | 0.66 | 1.91E-03 |
| cg09449543 | AUTS2 | Body | TRUE | 0 | 7 | 69246130 | 0.67 | 0.61 | 0.66 | 2.08E-03 |
