# Supplementary material for: REC8 is a novel tumor suppressor gene epigenetically robustly targeted by the PI3K pathway in thyroid cancer
Source: Oncotarget. 2015 Oct 13;6(36):39211–24. doi: 10.18632/oncotarget.5391 (PMC4770767; doi:10.18632/oncotarget.5391)
Supplement: Supplementary file 3 [file oncotarget-06-39211-s003.docx]

**Supplementary Table 2. Gene genetic/epigenetic analysis results for the thyroid samples using in this study**

| Sample number | Sample pathology | Matched sample | NRAS mutation | KRAS mutation | HRAS mutation | PIK3CA mutation | PTEN mutation | REC8 methylation | ADREB2B methylation |
| --- | --- | --- | --- | --- | --- | --- | --- | --- | --- |
| 1 | Normal | M10 | NA | NA | NA | NA | NA | 339.08 | 1664.51 |
| 2 | Normal | M9 | NA | NA | NA | NA | NA | 588.23 | 1733.53 |
| 3 | Normal | M6 | NA | NA | NA | NA | NA | 30.17 | 612.85 |
| 4 | Normal |  | NA | NA | NA | NA | NA | 482.48 | 1856.33 |
| 5 | Normal | M8 | NA | NA | NA | NA | NA | 277.21 | 1192.89 |
| 6 | Normal |  | NA | NA | NA | NA | NA | 361.1 | 976.56 |
| 7 | Normal |  | NA | NA | NA | NA | NA | 181.41 | 246.05 |
| 8 | Normal | M5 | NA | NA | NA | NA | NA | 114.13 | 829.26 |
| 9 | Normal |  | NA | NA | NA | NA | NA | 312.8 | 690.95 |
| 10 | Normal | M7 | NA | NA | NA | NA | NA | 259.71 | 1146.8 |
| 11 | Normal | M11 | NA | NA | NA | NA | NA | 536.16 | 3957.52 |
| 12 | Normal |  | NA | NA | NA | NA | NA | 591.52 | 1819.57 |
| 13 | Normal | M12 | NA | NA | NA | NA | NA | 291.01 | 1167.54 |
| 14 | Normal | M14 | NA | NA | NA | NA | NA | 249.05 | 597.68 |
| 15 | Normal | M1 | NA | NA | NA | NA | NA | 136.13 | 726.39 |
| 16 | Normal |  | NA | NA | NA | NA | NA | 0 | 8812.43 |
| 17 | Normal | M2 | NA | NA | NA | NA | NA | 265.08 | 753.57 |
| 18 | Normal | M16 | NA | NA | NA | NA | NA | 191.54 | 223.97 |
| 19 | Normal |  | NA | NA | NA | NA | NA | 487.03 | 830.84 |
| 20 | Normal |  | NA | NA | NA | NA | NA | 52.55 | 468.95 |
| 21 | Normal |  | NA | NA | NA | NA | NA | 251.48 | 500.21 |
| 22 | Normal |  | NA | NA | NA | NA | NA | 263.1 | 1244.8 |
| 23 | Normal | M3 | NA | NA | NA | NA | NA | 460.68 | 1253.43 |
| 24 | Normal | M4 | NA | NA | NA | NA | NA | 214.38 | 439.14 |
| 25 | Normal |  | NA | NA | NA | NA | NA | 49.1 | 430.51 |
| 26 | Benign |  | WT | WT | WT | WT | WT | 893.74 | 2343.57 |
| 27 | Benign |  | WT | WT | WT | WT | WT | 623.3 | 1791.08 |
| 28 | Benign |  | WT | WT | WT | WT | WT | 908.35 | 3562.65 |
| 29 | Benign |  | WT | WT | WT | WT | WT | 806.27 | 872.81 |
| 30 | Benign |  | WT | WT | WT | WT | WT | 497.2 | 5142.5 |
| 31 | Benign |  | WT | WT | WT | WT | WT | 547.95 | 344.95 |
| 32 | Benign |  | WT | WT | WT | WT | WT | 587.78 | 139.21 |
| 33 | Benign |  | WT | WT | WT | WT | WT | 468.46 | 2075.36 |
| 34 | Benign |  | WT | WT | WT | WT | WT | 244.81 | 605.77 |
| 35 | Benign |  | WT | WT | WT | WT | WT | 1062.53 | 716.09 |
| 36 | Benign |  | WT | WT | WT | WT | WT | 538.77 | 102.24 |
| 37 | Benign |  | WT | WT | WT | WT | WT | 433.44 | 692.43 |
| 38 | Benign |  | WT | WT | WT | WT | WT | 717.01 | 204.22 |
| 39 | Benign |  | WT | WT | WT | WT | WT | 248.37 | 724.71 |
| 40 | Benign |  | WT | WT | WT | WT | WT | 269.32 | 333.54 |
| 41 | Benign |  | WT | WT | WT | WT | WT | 461.84 | 892.82 |
| 42 | Benign |  | WT | WT | WT | WT | WT | 739.5 | 2648.25 |
| 43 | Benign |  | Q61K | WT | WT | WT | WT | 516.37 | 990.87 |
| 44 | Benign |  | NA | WT | WT | NA | NA | 403.16 | 3381.41 |
| 45 | Benign |  | NA | WT | WT | NA | NA | 672.36 | 708.16 |
| 46 | PTC | M12 | WT | WT | WT | WT | R142Q | 294.29 | 1977.4 |
| 47 | PTC | M9 | WT | WT | WT | WT | WT | 556.45 | 1082.18 |
| 48 | PTC |  | WT | WT | WT | WT | WT | 1166.21 | 667.51 |
| 49 | PTC |  | WT | WT | WT | WT | WT | 987.69 | 1811.65 |
| 50 | PTC |  | WT | WT | WT | WT | WT | 458.73 | 324.57 |
| 51 | PTC |  | WT | WT | WT | WT | WT | 153.86 | 1458.69 |
| 52 | PTC |  | WT | WT | WT | WT | WT | 367.69 | 2965.89 |
| 53 | PTC | M11 | WT | WT | WT | WT | WT | 871.37 | 2709.52 |
| 54 | PTC |  | WT | WT | WT | WT | WT | 517.9 | 330.04 |
| 55 | PTC |  | WT | WT | WT | WT | WT | 578.21 | 2218.45 |
| 56 | PTC |  | WT | WT | WT | WT | WT | 337.8 | 628.01 |
| 57 | PTC | M6 | WT | WT | WT | WT | WT | 54.21 | 129.86 |
| 58 | PTC | M5 | WT | WT | WT | WT | WT | 172.29 | 101.89 |
| 59 | PTC |  | WT | WT | WT | WT | WT | 562.27 | 2719.83 |
| 60 | PTC |  | WT | WT | WT | WT | WT | 1165.35 | 1710.17 |
| 61 | PTC |  | WT | WT | WT | WT | WT | 1122.64 | 1891.14 |
| 62 | PTC |  | WT | WT | WT | WT | WT | 625.85 | 1302.78 |
| 63 | PTC |  | WT | WT | WT | WT | WT | 889.87 | 2243.57 |
| 64 | PTC |  | WT | WT | WT | WT | WT | 767.56 | 1990.33 |
| 65 | PTC |  | WT | WT | WT | WT | WT | 843.15 | 1287.4 |
| 66 | PTC |  | WT | WT | WT | WT | WT | 465.55 | 822.21 |
| 67 | PTC | M8 | WT | WT | WT | WT | WT | 345.39 | 2935.76 |
| 68 | PTC |  | WT | WT | WT | WT | WT | 235.01 | 166.97 |
| 69 | PTC | M7 | WT | WT | WT | WT | WT | 821.01 | 3637.41 |
| 70 | PTC | M17 | WT | WT | WT | WT | WT | 412.54 | 861.43 |
| 71 | PTC | M13 | WT | WT | WT | WT | WT | 192.59 | 185.6 |
| 72 | PTC |  | WT | WT | WT | WT | WT | 183.47 | 235.86 |
| 73 | PTC |  | WT | WT | WT | S553N | WT | 349.34 | 1166.09 |
| 74 | PTC |  | WT | WT | WT | I1062V | WT | 531.05 | 2006.73 |
| 75 | PTC |  | Q61R | WT | WT | WT | WT | 780.26 | 0 |
| 76 | PTC |  | Q61R | WT | WT | WT | WT | 144.18 | 977.29 |
| 77 | PTC |  | NA | WT | WT | NA | NA | 473.19 | 1798.69 |
| 78 | PTC |  | NA | WT | WT | NA | NA | 495.27 | 2052.69 |
| 79 | PTC | M15 | WT | WT | WT | WT | WT | 232.48 | 555.52 |
| 80 | PTC |  | WT | WT | WT | WT | WT | 858.85 | 509.63 |
| 81 | PTC |  | WT | WT | WT | WT | WT | 480.88 | 1944.45 |
| 82 | PTC | M10 | WT | WT | WT | WT | WT | 475.91 | 1805.91 |
| 83 | PTC |  | WT | WT | WT | WT | WT | 858.4 | 991.78 |
| 84 | PTC |  | WT | WT | WT | F1039L | WT | 518.29 | 465.47 |
| 85 | FTC |  | WT | WT | WT | WT | R130Q | 443.2 | 1046.21 |
| 86 | FTC | M2 | WT | WT | WT | WT | WT | 267.58 | 252.61 |
| 87 | FTC |  | WT | WT | WT | WT | WT | 503.48 | 2329.37 |
| 88 | FTC |  | WT | WT | WT | WT | WT | 516.29 | 677.09 |
| 89 | FTC |  | WT | WT | WT | WT | WT | 2324.68 | 2052.74 |
| 90 | FTC |  | WT | WT | WT | WT | WT | 1271.7 | 1559.86 |
| 91 | FTC |  | WT | WT | WT | WT | WT | 927.84 | 482.07 |
| 92 | FTC |  | WT | WT | WT | WT | WT | 1101.18 | 377.06 |
| 93 | FTC |  | WT | WT | WT | WT | WT | 780.74 | 1813.8 |
| 94 | FTC |  | WT | WT | WT | WT | WT | 748.67 | 2284.64 |
| 95 | FTC |  | WT | WT | WT | WT | WT | 202.76 | 2888.79 |
| 96 | FTC |  | WT | WT | WT | WT | WT | 130.04 | 543.21 |
| 97 | FTC |  | WT | WT | WT | WT | WT | 501.89 | 302.11 |
| 98 | FTC |  | WT | WT | WT | WT | WT | 289.05 | 1049.08 |
| 99 | FTC |  | WT | WT | WT | WT | WT | 842.5 | 130.38 |
| 100 | FTC |  | WT | WT | WT | WT | WT | 770.31 | 643.94 |
| 101 | FTC |  | WT | WT | WT | WT | WT | 268.48 | 1176.32 |
| 102 | FTC |  | WT | WT | WT | WT | WT | 924.23 | 1347.62 |
| 103 | FTC |  | WT | WT | WT | WT | WT | 451.53 | 2032.61 |
| 104 | FTC |  | WT | WT | WT | WT | WT | 653.31 | 977.46 |
| 105 | FTC |  | WT | WT | WT | WT | WT | 1260.65 | 1897.62 |
| 106 | FTC |  | WT | WT | WT | WT | WT | 1041.92 | 3008.57 |
| 107 | FTC |  | WT | WT | WT | WT | WT | 818.02 | 1587.8 |
| 108 | FTC |  | WT | WT | WT | WT | WT | 187.07 | 1070.23 |
| 109 | FTC |  | WT | WT | WT | WT | WT | 822.81 | 3385.33 |
| 110 | FTC |  | WT | WT | WT | WT | WT | 334.15 | 1216.27 |
| 111 | FTC |  | WT | WT | WT | WT | WT | 1069.11 | 3832.37 |
| 112 | FTC |  | WT | WT | WT | WT | WT | 1160.16 | 878.67 |
| 113 | FTC |  | WT | WT | WT | WT | WT | 589.49 | 589.49 |
| 114 | FTC |  | WT | WT | WT | WT | WT | 135.67 | 1691.01 |
| 115 | FTC |  | WT | WT | WT | WT | WT | 284.27 | 510 |
| 116 | FTC |  | WT | WT | WT | WT | WT | 59.66 | 284.84 |
| 117 | FTC |  | WT | WT | WT | WT | WT | 404.54 | 568.66 |
| 118 | FTC |  | WT | WT | WT | WT | WT | 356.04 | 998.64 |
| 119 | FTC |  | WT | WT | WT | WT | WT | 1194.57 | 262.39 |
| 120 | FTC |  | WT | WT | WT | WT | WT | 989.03 | 1289.31 |
| 121 | FTC |  | WT | WT | WT | WT | WT | 388.63 | 859.05 |
| 122 | FTC |  | WT | WT | WT | WT | WT | 632.44 | 1809.52 |
| 123 | FTC |  | WT | WT | WT | WT | WT | 508.39 | 2225 |
| 124 | FTC |  | WT | WT | WT | WT | WT | 338.84 | 1106.98 |
| 125 | FTC |  | WT | WT | WT | WT | WT | 426.22 | 650.69 |
| 126 | FTC |  | WT | WT | WT | WT | WT | 418.63 | 290.78 |
| 127 | FTC |  | WT | WT | WT | WT | WT | 449.48 | 1015.95 |
| 128 | FTC | M3 | WT | WT | WT | WT | WT | 408.63 | 844.2 |
| 129 | FTC | M4 | WT | WT | WT | WT | WT | 568.91 | 604.22 |
| 130 | FTC |  | WT | WT | WT | WT | WT | 469.08 | 1196.28 |
| 131 | FTC |  | WT | WT | WT | E545A | R161G | 464.35 | 239.21 |
| 132 | FTC |  | WT | WT | WT | D520N | WT | 1114.95 | 1929.54 |
| 133 | FTC |  | Q61R | WT | WT | WT | R159K | 1038.16 | 2215.14 |
| 134 | FTC | M1 | Q61R | WT | WT | WT | WT | 235.7 | 1044.6 |
| 135 | FTC |  | Q61R | WT | WT | WT | WT | 430.58 | 966.54 |
| 136 | FTC |  | Q61R | WT | WT | WT | WT | 809.93 | 662.24 |
| 137 | FTC |  | Q61R | WT | WT | WT | WT | 1020.69 | 1550.77 |
| 138 | FTC |  | Q61R | WT | WT | WT | WT | 606.47 | 1571.99 |
| 139 | FTC |  | Q61R | WT | WT | WT | WT | 1287.21 | 2921.85 |
| 140 | FTC |  | Q61R | WT | WT | WT | WT | 1514.77 | 5149.11 |
| 141 | FTC |  | Q61R | WT | WT | L1001I | WT | 491.76 | 1968.7 |
| 142 | FTC |  | Q61K | WT | WT | WT | WT | 568.26 | 1186.42 |
| 143 | FTC |  | Q61K | WT | WT | WT | WT | 849.31 | 3094.82 |
| 144 | FTC |  | WT | G12R | WT | WT | WT | 654.65 | 2724.15 |
| 145 | ATC |  | WT | WT | WT | WT | D162G | 1358.37 | 5496.5 |
| 146 | ATC |  | WT | WT | WT | WT | K147R | 3318.03 | 2360.62 |
| 147 | ATC |  | WT | WT | WT | WT | P226L | 1094.89 | 1232.42 |
| 148 | ATC |  | WT | WT | WT | WT | WT | 737.98 | 4857.96 |
| 149 | ATC |  | WT | WT | WT | WT | WT | 1115.61 | 4744.12 |
| 150 | ATC |  | WT | WT | WT | WT | WT | 149.7 | 739.97 |
| 151 | ATC |  | WT | WT | WT | WT | WT | 674.61 | 2247.46 |
| 152 | ATC |  | WT | WT | WT | WT | WT | 140.32 | 341.63 |
| 153 | ATC | M17 | WT | WT | WT | WT | WT | 513.96 | 123.47 |
| 154 | ATC | M15 | WT | WT | WT | WT | WT | 951.59 | 2105.49 |
| 155 | ATC |  | WT | WT | WT | WT | WT | 910.59 | 2698.09 |
| 156 | ATC |  | WT | WT | WT | WT | WT | 851.08 | 1781.99 |
| 157 | ATC |  | WT | WT | WT | WT | WT | 805.29 | 1542.99 |
| 158 | ATC |  | WT | WT | WT | WT | WT | 849.19 | 1702.45 |
| 159 | ATC |  | WT | WT | WT | WT | WT | 461.46 | 5358.31 |
| 160 | ATC |  | WT | WT | WT | WT | WT | 468.88 | 1176.37 |
| 161 | ATC |  | WT | WT | WT | WT | WT | 497.39 | 5849.75 |
| 162 | ATC |  | WT | WT | WT | WT | WT | 976.31 | 2130.16 |
| 163 | ATC |  | WT | WT | WT | WT | WT | 862.62 | 4445.49 |
| 164 | ATC |  | WT | WT | WT | WT | WT | 773.24 | 1130.19 |
| 165 | ATC |  | WT | WT | WT | WT | WT | 1373.84 | 750.87 |
| 166 | ATC |  | WT | WT | WT | WT | WT | 203.9 | 777.39 |
| 167 | ATC |  | WT | WT | WT | WT | WT | 609.14 | 933.9 |
| 168 | ATC | M13 | WT | WT | WT | WT | WT | 903.94 | 2662.56 |
| 169 | ATC |  | WT | WT | WT | WT | WT | 979.51 | 2938.55 |
| 170 | ATC | M14 | WT | WT | WT | WT | WT | 1319.6 | 866.58 |
| 171 | ATC |  | WT | WT | WT | WT | WT | 857.06 | 2197.16 |
| 172 | ATC |  | WT | WT | WT | WT | WT | 763.5 | 1113.93 |
| 173 | ATC |  | WT | WT | WT | WT | WT | 756.14 | 2662.24 |
| 174 | ATC |  | WT | WT | WT | WT | WT | 721.61 | 1653.36 |
| 175 | ATC |  | WT | WT | WT | WT | WT | 444.37 | 1809.51 |
| 176 | ATC |  | WT | WT | WT | WT | WT | 394.01 | 1133.36 |
| 177 | ATC |  | WT | WT | WT | WT | WT | 583.59 | 1290.12 |
| 178 | ATC | M16 | WT | WT | WT | WT | WT | 354.2 | 1507 |
| 179 | ATC |  | WT | WT | WT | WT | WT | 1074.83 | 283.7 |
| 180 | ATC |  | Q61R | WT | WT | WT | WT | 344.46 | 54.05 |
| 181 | ATC |  | Q61R | WT | WT | H1047L | WT | 565.98 | 510.42 |
| 182 | ATC |  | Q61K | WT | WT | WT | WT | 1083.53 | 154.1 |
