# Supplementary material for: REC8 is a novel tumor suppressor gene epigenetically robustly targeted by the PI3K pathway in thyroid cancer
Source: Oncotarget. 2015 Oct 13;6(36):39211–24. doi: 10.18632/oncotarget.5391 (PMC4770767; doi:10.18632/oncotarget.5391)
Supplement: Supplementary file 4 [file oncotarget-06-39211-s004.docx]

**Supplementary Table 3. REC8 mRNA level and predicted PI3K pathway activity for individual colon cancer or melanoma samples**

| Sample | REC8 level | PI3K pathway activity | Sample | REC8 level | PI3K pathway activity |
| --- | --- | --- | --- | --- | --- |
| Colon cancer | | | melanoma | | |
| GSM972381 | 68.73609 | 0.049259 | GSM259617 | 91.66008 | 0.120484 |
| GSM972190 | 93.05412 | 0.06764 | GSM259618 | 78.80451 | 0.13431 |
| GSM972424 | 103.0603 | 0.07303 | GSM259631 | 98.93128 | 0.159495 |
| GSM972034 | 138.9229 | 0.083975 | GSM276745 | 128.0405 | 0.180924 |
| GSM972024 | 89.17333 | 0.097297 | GSM276756 | 138.4112 | 0.184435 |
| GSM972267 | 98.86608 | 0.099524 | GSM390235 | 168.8495 | 0.191122 |
| GSM972121 | 143.6201 | 0.099534 | GSM259645 | 74.416 | 0.191821 |
| GSM972097 | 134.4335 | 0.101557 | GSM276752 | 79.72315 | 0.20491 |
| GSM972139 | 83.56786 | 0.101599 | GSM259644 | 106.4537 | 0.209944 |
| GSM972018 | 247.8316 | 0.116203 | GSM276759 | 302.6008 | 0.211762 |
| GSM972183 | 144.333 | 0.117754 | GSM259630 | 93.77056 | 0.213759 |
| GSM972017 | 166.6654 | 0.120173 | GSM276755 | 493.0683 | 0.21379 |
| GSM972295 | 112.5808 | 0.141933 | GSM390265 | 116.6159 | 0.23139 |
| GSM972096 | 140.3072 | 0.141963 | GSM390240 | 113.3191 | 0.241215 |
| GSM972297 | 125.5638 | 0.143305 | GSM833481 | 201.9929 | 0.241355 |
| GSM972127 | 89.02606 | 0.145579 | GSM390257 | 152.3258 | 0.24164 |
| GSM972288 | 129.098 | 0.146325 | GSM259629 | 116.209 | 0.243091 |
| GSM972185 | 77.05914 | 0.150671 | GSM259619 | 67.75984 | 0.263049 |
| GSM972394 | 87.73433 | 0.156229 | GSM563419 | 183.7241 | 0.269913 |
| GSM972502 | 109.1995 | 0.158274 | GSM390254 | 198.3732 | 0.272997 |
| GSM972113 | 74.34961 | 0.159199 | GSM276747 | 93.8677 | 0.274441 |
| GSM972428 | 118.4894 | 0.159448 | GSM700750 | 103.6025 | 0.281635 |
| GSM972030 | 91.08418 | 0.159522 | GSM276725 | 118.6627 | 0.287333 |
| GSM972282 | 135.2776 | 0.160551 | GSM259635 | 441.546 | 0.288011 |
| GSM972188 | 84.51595 | 0.161089 | GSM276757 | 191.3144 | 0.291714 |
| GSM972283 | 251.3802 | 0.162873 | GSM390255 | 183.5312 | 0.299884 |
| GSM972416 | 77.73117 | 0.164438 | GSM563414 | 111.5832 | 0.300282 |
| GSM972259 | 162.6643 | 0.16686 | GSM390252 | 189.9022 | 0.301885 |
| GSM972412 | 103.5305 | 0.170844 | GSM390232 | 233.8661 | 0.319466 |
| GSM972292 | 135.7659 | 0.18002 | GSM390238 | 135.5838 | 0.319843 |
| GSM972270 | 91.19039 | 0.180373 | GSM259625 | 84.88463 | 0.320456 |
| GSM972175 | 69.73623 | 0.182293 | GSM259634 | 298.4581 | 0.322706 |
| GSM972254 | 88.05947 | 0.185924 | GSM563408 | 132.0064 | 0.325706 |
| GSM972023 | 111.491 | 0.1864 | GSM276744 | 65.77462 | 0.327135 |
| GSM972273 | 121.996 | 0.188344 | GSM276751 | 78.23168 | 0.329981 |
| GSM972100 | 114.6981 | 0.190525 | GSM276761 | 116.4476 | 0.33722 |
| GSM972029 | 135.2421 | 0.191844 | GSM276727 | 111.3816 | 0.338177 |
| GSM972111 | 84.71403 | 0.192437 | GSM563417 | 420.7716 | 0.354986 |
| GSM972503 | 102.5171 | 0.195451 | GSM259627 | 111.7992 | 0.358604 |
| GSM972274 | 261.7875 | 0.195771 | GSM833483 | 74.86796 | 0.363197 |
| GSM972228 | 121.2397 | 0.197414 | GSM276762 | 123.5532 | 0.36567 |
| GSM972172 | 99.40094 | 0.200767 | GSM259626 | 179.8029 | 0.367425 |
| GSM972019 | 204.3465 | 0.201589 | GSM390237 | 230.6689 | 0.368565 |
| GSM972390 | 96.90734 | 0.206601 | GSM390264 | 420.8211 | 0.369487 |
| GSM972275 | 127.9516 | 0.210083 | GSM390253 | 161.5184 | 0.371455 |
| GSM972192 | 94.38744 | 0.212025 | GSM259659 | 173.5528 | 0.381353 |
| GSM972099 | 78.81951 | 0.212194 | GSM700744 | 125.2333 | 0.38936 |
| GSM972258 | 94.32504 | 0.213048 | GSM390276 | 100.285 | 0.389585 |
| GSM972098 | 165.9012 | 0.214503 | GSM276754 | 119.1686 | 0.396616 |
| GSM972269 | 95.1973 | 0.216098 | GSM276743 | 72.05054 | 0.400093 |
| GSM972500 | 251.1453 | 0.220693 | GSM259628 | 103.3241 | 0.40381 |
| GSM972418 | 110.9615 | 0.222711 | GSM390230 | 247.2717 | 0.418136 |
| GSM972501 | 81.18805 | 0.22291 | GSM276726 | 107.9009 | 0.419627 |
| GSM972518 | 103.4851 | 0.223755 | GSM259656 | 62.86625 | 0.421736 |
| GSM972028 | 163.9266 | 0.226668 | GSM390267 | 211.3904 | 0.427461 |
| GSM972244 | 125.1505 | 0.227512 | GSM390245 | 60.38251 | 0.429682 |
| GSM972149 | 98.2208 | 0.227588 | GSM390261 | 41.68035 | 0.441411 |
| GSM972443 | 90.77999 | 0.228071 | GSM259658 | 109.4741 | 0.441952 |
| GSM972516 | 157.9321 | 0.229123 | GSM276730 | 102.0413 | 0.443708 |
| GSM972391 | 107.4726 | 0.229384 | GSM390243 | 147.4214 | 0.444047 |
| GSM972116 | 103.469 | 0.230422 | GSM390251 | 107.8252 | 0.448392 |
| GSM972247 | 125.0605 | 0.230624 | GSM259646 | 110.402 | 0.450409 |
| GSM972130 | 167.9006 | 0.231752 | GSM390281 | 69.05112 | 0.450787 |
| GSM972260 | 116.9514 | 0.234938 | GSM276748 | 90.67252 | 0.454402 |
| GSM972245 | 153.9791 | 0.235161 | GSM259621 | 92.87395 | 0.457123 |
| GSM972173 | 93.69341 | 0.239217 | GSM276764 | 60.70611 | 0.457515 |
| GSM972299 | 142.4703 | 0.240874 | GSM390258 | 84.68581 | 0.458414 |
| GSM972105 | 258.1483 | 0.242945 | GSM563416 | 89.80373 | 0.459177 |
| GSM972413 | 74.95226 | 0.244882 | GSM390256 | 191.8485 | 0.459568 |
| GSM972027 | 105.1223 | 0.246132 | GSM276758 | 106.3368 | 0.460927 |
| GSM972357 | 85.31617 | 0.246661 | GSM563420 | 132.1135 | 0.461325 |
| GSM972199 | 92.18123 | 0.24967 | GSM700747 | 100.9648 | 0.462628 |
| GSM972396 | 93.74459 | 0.249798 | GSM563407 | 114.7191 | 0.464648 |
| GSM972253 | 116.247 | 0.253846 | GSM276737 | 140.1209 | 0.467006 |
| GSM972039 | 134.5944 | 0.254541 | GSM259657 | 44.80767 | 0.46704 |
| GSM972420 | 133.7158 | 0.256957 | GSM276738 | 111.9538 | 0.468317 |
| GSM972379 | 112.6608 | 0.257346 | GSM276760 | 120.1516 | 0.476259 |
| GSM972126 | 133.5371 | 0.258134 | GSM259649 | 135.077 | 0.476871 |
| GSM972219 | 103.6357 | 0.258767 | GSM276728 | 76.61191 | 0.478055 |
| GSM972181 | 97.87065 | 0.260783 | GSM563410 | 103.651 | 0.479565 |
| GSM972118 | 90.15095 | 0.26521 | GSM700746 | 90.00726 | 0.48536 |
| GSM972505 | 83.45612 | 0.266242 | GSM390246 | 150.9651 | 0.485464 |
| GSM972263 | 128.1549 | 0.266278 | GSM700751 | 157.5662 | 0.500755 |
| GSM972522 | 110.9615 | 0.268613 | GSM276742 | 112.9914 | 0.501588 |
| GSM972174 | 107.1955 | 0.268718 | GSM390260 | 137.6164 | 0.501701 |
| GSM972102 | 152.9047 | 0.270379 | GSM390247 | 238.7256 | 0.503792 |
| GSM972179 | 109.2893 | 0.270955 | GSM390250 | 158.6606 | 0.504025 |
| GSM972375 | 109.0831 | 0.272326 | GSM563409 | 124.432 | 0.505449 |
| GSM972367 | 117.6338 | 0.272491 | GSM700743 | 106.1386 | 0.506878 |
| GSM972519 | 89.76455 | 0.27292 | GSM259623 | 131.5687 | 0.513613 |
| GSM972251 | 123.583 | 0.273746 | GSM276772 | 114.9929 | 0.516119 |
| GSM972186 | 95.46541 | 0.275316 | GSM390269 | 115.9246 | 0.516963 |
| GSM972257 | 112.906 | 0.276607 | GSM390268 | 93.74057 | 0.521663 |
| GSM972392 | 103.2486 | 0.277467 | GSM390224 | 76.59426 | 0.522741 |
| GSM972036 | 169.7123 | 0.277775 | GSM276735 | 79.53165 | 0.525618 |
| GSM972177 | 94.99746 | 0.277858 | GSM276739 | 64.80035 | 0.526328 |
| GSM972276 | 129.0267 | 0.278376 | GSM259632 | 117.2582 | 0.529195 |
| GSM972022 | 155.7758 | 0.27971 | GSM390279 | 109.1628 | 0.53059 |
| GSM972415 | 75.88934 | 0.280142 | GSM700742 | 114.4667 | 0.536566 |
| GSM972000 | 75.94863 | 0.280772 | GSM390263 | 141.1552 | 0.537713 |
| GSM971986 | 161.8819 | 0.280969 | GSM833482 | 111.1609 | 0.541795 |
| GSM972294 | 229.3158 | 0.283587 | GSM390272 | 89.2827 | 0.545008 |
| GSM972232 | 130.6596 | 0.288109 | GSM390280 | 133.5904 | 0.545187 |
| GSM972429 | 101.5361 | 0.288575 | GSM390278 | 113.9513 | 0.546012 |
| GSM972373 | 74.09338 | 0.288702 | GSM259650 | 157.137 | 0.546913 |
| GSM972156 | 80.67766 | 0.291029 | GSM276749 | 138.25 | 0.553104 |
| GSM972271 | 114.5795 | 0.291565 | GSM259647 | 76.03616 | 0.553226 |
| GSM972446 | 100.2851 | 0.294023 | GSM259633 | 113.1655 | 0.556602 |
| GSM972226 | 100.7248 | 0.295234 | GSM276732 | 77.03278 | 0.558513 |
| GSM972441 | 118.9998 | 0.295862 | GSM259648 | 76.02136 | 0.560955 |
| GSM972140 | 96.94861 | 0.296186 | GSM390229 | 250.9718 | 0.562848 |
| GSM972224 | 169.9205 | 0.297271 | GSM390270 | 49.88995 | 0.565232 |
| GSM972059 | 114.4253 | 0.297633 | GSM563411 | 153.0776 | 0.56571 |
| GSM972150 | 90.40637 | 0.297756 | GSM390226 | 118.0582 | 0.568594 |
| GSM972426 | 110.7991 | 0.297779 | GSM259640 | 90.66041 | 0.570321 |
| GSM972427 | 87.77749 | 0.298193 | GSM259641 | 167.7631 | 0.574852 |
| GSM972256 | 89.99293 | 0.29936 | GSM700753 | 95.80862 | 0.57845 |
| GSM972378 | 128.8711 | 0.300199 | GSM276723 | 191.7202 | 0.578755 |
| GSM972144 | 81.3711 | 0.302663 | GSM700749 | 120.1651 | 0.580002 |
| GSM972246 | 82.44386 | 0.303001 | GSM259622 | 88.18582 | 0.58111 |
| GSM972284 | 98.2608 | 0.307018 | GSM276734 | 137.9206 | 0.582949 |
| GSM972195 | 115.2132 | 0.314074 | GSM276770 | 125.8016 | 0.591853 |
| GSM972265 | 125.0377 | 0.316023 | GSM390273 | 116.0874 | 0.591862 |
| GSM972031 | 123.8369 | 0.316348 | GSM563412 | 98.21928 | 0.591984 |
| GSM972182 | 71.63806 | 0.31664 | GSM563413 | 119.4198 | 0.592091 |
| GSM972223 | 84.0843 | 0.317003 | GSM563418 | 125.9113 | 0.592318 |
| GSM972191 | 97.5223 | 0.317066 | GSM259620 | 64.206 | 0.593063 |
| GSM972180 | 202.4692 | 0.317748 | GSM700745 | 92.57755 | 0.59941 |
| GSM972262 | 152.69 | 0.318676 | GSM276724 | 85.41312 | 0.599544 |
| GSM972153 | 84.24759 | 0.319405 | GSM700752 | 84.72841 | 0.604099 |
| GSM972510 | 118.8344 | 0.319683 | GSM390244 | 173.7979 | 0.604333 |
| GSM972248 | 105.3311 | 0.320285 | GSM390241 | 37.56042 | 0.606224 |
| GSM972272 | 100.973 | 0.321375 | GSM390266 | 172.035 | 0.609407 |
| GSM972506 | 70.05858 | 0.3231 | GSM276731 | 112.9009 | 0.611797 |
| GSM971996 | 291.5401 | 0.324695 | GSM276771 | 110.4483 | 0.61511 |
| GSM972026 | 100.6577 | 0.326779 | GSM390275 | 64.39162 | 0.617016 |
| GSM972163 | 95.47983 | 0.327946 | GSM390233 | 222.561 | 0.625631 |
| GSM972077 | 87.35343 | 0.33064 | GSM390277 | 145.8943 | 0.630507 |
| GSM972304 | 100.4467 | 0.330732 | GSM390225 | 139.4135 | 0.632489 |
| GSM972033 | 82.06562 | 0.331791 | GSM276753 | 100.3893 | 0.63784 |
| GSM972161 | 94.4251 | 0.332848 | GSM259624 | 97.59899 | 0.641522 |
| GSM972107 | 97.02654 | 0.333334 | GSM390248 | 111.798 | 0.641883 |
| GSM972249 | 99.66649 | 0.334345 | GSM390231 | 97.08085 | 0.642789 |
| GSM972160 | 126.3936 | 0.334862 | GSM259638 | 82.93973 | 0.647645 |
| GSM972061 | 80.72717 | 0.335439 | GSM276746 | 77.2566 | 0.650659 |
| GSM972301 | 110.7064 | 0.336724 | GSM276768 | 107.4152 | 0.653028 |
| GSM972079 | 85.03945 | 0.337984 | GSM276740 | 93.81302 | 0.653064 |
| GSM972204 | 106.1562 | 0.339121 | GSM390262 | 158.4239 | 0.653988 |
| GSM972122 | 109.3697 | 0.339763 | GSM390228 | 98.56313 | 0.654495 |
| GSM972211 | 80.75297 | 0.339895 | GSM259643 | 168.8446 | 0.655189 |
| GSM972504 | 104.7416 | 0.340383 | GSM390249 | 192.7182 | 0.655996 |
| GSM972133 | 98.05006 | 0.341871 | GSM276769 | 74.58564 | 0.65677 |
| GSM972170 | 89.22324 | 0.342115 | GSM276733 | 85.36313 | 0.657259 |
| GSM972006 | 93.56585 | 0.343006 | GSM390239 | 136.021 | 0.665401 |
| GSM972151 | 93.18313 | 0.345473 | GSM390271 | 125.0381 | 0.665403 |
| GSM972393 | 110.5442 | 0.345846 | GSM390242 | 250.3767 | 0.665515 |
| GSM972311 | 78.80758 | 0.345887 | GSM259637 | 78.9631 | 0.667038 |
| GSM972145 | 93.21147 | 0.348018 | GSM390236 | 109.3529 | 0.66866 |
| GSM972162 | 94.02899 | 0.350464 | GSM390227 | 103.9928 | 0.673349 |
| GSM972417 | 75.10782 | 0.350998 | GSM700748 | 94.69345 | 0.673644 |
| GSM972187 | 95.60526 | 0.354125 | GSM276741 | 105.5225 | 0.676172 |
| GSM972148 | 115.0349 | 0.3576 | GSM276763 | 70.294 | 0.679657 |
| GSM972157 | 94.22343 | 0.358961 | GSM276729 | 74.03128 | 0.693111 |
| GSM972115 | 102.7261 | 0.360471 | GSM390274 | 128.7342 | 0.695993 |
| GSM972057 | 60.55033 | 0.360789 | GSM259636 | 74.17064 | 0.730225 |
| GSM972125 | 108.1485 | 0.364253 | GSM390259 | 174.3366 | 0.732234 |
| GSM972240 | 158.138 | 0.367221 | GSM276750 | 146.9084 | 0.737266 |
| GSM972025 | 111.6232 | 0.367518 | GSM390234 | 57.93257 | 0.741904 |
| GSM972123 | 98.47083 | 0.368879 | GSM276736 | 108.5311 | 0.752191 |
| GSM972430 | 93.81405 | 0.371353 | GSM259652 | 87.97815 | 0.765309 |
| GSM972178 | 122.0972 | 0.371452 | GSM259655 | 101.5373 | 0.801695 |
| GSM972138 | 124.9449 | 0.371817 | GSM563415 | 84.97848 | 0.811343 |
| GSM972422 | 88.86763 | 0.372098 | GSM259642 | 97.04773 | 0.812955 |
| GSM972146 | 81.74815 | 0.372903 | GSM259639 | 66.51136 | 0.814686 |
| GSM972425 | 89.89281 | 0.373375 | GSM259651 | 145.8782 | 0.820395 |
| GSM972184 | 85.10241 | 0.374338 | GSM276766 | 78.40123 | 0.823733 |
| GSM972169 | 95.30718 | 0.374783 | GSM276767 | 119.6696 | 0.825258 |
| GSM972135 | 92.39933 | 0.376071 | GSM276765 | 75.37463 | 0.837191 |
| GSM972216 | 106.49 | 0.376543 | GSM259653 | 102.1036 | 0.844678 |
| GSM972436 | 98.98621 | 0.378687 | GSM259654 | 79.34228 | 0.862143 |
| GSM972300 | 97.39507 | 0.38052 |  |  |  |
| GSM972280 | 92.61329 | 0.383987 |  |  |  |
| GSM971995 | 116.196 | 0.384337 |  |  |  |
| GSM972176 | 108.3789 | 0.384894 |  |  |  |
| GSM972279 | 93.52897 | 0.3858 |  |  |  |
| GSM972171 | 141.6494 | 0.387896 |  |  |  |
| GSM972003 | 92.00851 | 0.388012 |  |  |  |
| GSM972399 | 119.2306 | 0.389789 |  |  |  |
| GSM972277 | 78.97101 | 0.392082 |  |  |  |
| GSM972255 | 87.78001 | 0.393171 |  |  |  |
| GSM972290 | 110.2418 | 0.394748 |  |  |  |
| GSM972419 | 80.3836 | 0.395432 |  |  |  |
| GSM972136 | 86.2783 | 0.395781 |  |  |  |
| GSM972371 | 134.7551 | 0.395899 |  |  |  |
| GSM972011 | 161.4128 | 0.396921 |  |  |  |
| GSM972193 | 119.1823 | 0.397592 |  |  |  |
| GSM972241 | 118.7765 | 0.397948 |  |  |  |
| GSM972444 | 180.9016 | 0.400066 |  |  |  |
| GSM972291 | 136.7482 | 0.401843 |  |  |  |
| GSM972395 | 99.94699 | 0.401852 |  |  |  |
| GSM971991 | 103.7054 | 0.402257 |  |  |  |
| GSM972131 | 68.47625 | 0.406359 |  |  |  |
| GSM972296 | 168.9373 | 0.407672 |  |  |  |
| GSM972397 | 96.65062 | 0.408741 |  |  |  |
| GSM972293 | 118.4493 | 0.40921 |  |  |  |
| GSM972305 | 122.5066 | 0.411967 |  |  |  |
| GSM972421 | 96.67215 | 0.417277 |  |  |  |
| GSM971998 | 108.6999 | 0.417798 |  |  |  |
| GSM972227 | 115.3493 | 0.422027 |  |  |  |
| GSM972370 | 101.2804 | 0.422323 |  |  |  |
| GSM972101 | 99.59958 | 0.422615 |  |  |  |
| GSM972239 | 85.12455 | 0.4252 |  |  |  |
| GSM972205 | 133.3535 | 0.425451 |  |  |  |
| GSM972287 | 151.0036 | 0.426249 |  |  |  |
| GSM972423 | 87.92235 | 0.426608 |  |  |  |
| GSM972250 | 71.77154 | 0.427861 |  |  |  |
| GSM972382 | 64.81988 | 0.42801 |  |  |  |
| GSM972210 | 126.6795 | 0.428228 |  |  |  |
| GSM971989 | 88.09091 | 0.4302 |  |  |  |
| GSM972495 | 102.0578 | 0.431943 |  |  |  |
| GSM972366 | 79.38058 | 0.434781 |  |  |  |
| GSM972230 | 147.6705 | 0.435582 |  |  |  |
| GSM972093 | 78.48642 | 0.436606 |  |  |  |
| GSM972398 | 102.4407 | 0.43757 |  |  |  |
| GSM972217 | 97.03475 | 0.437689 |  |  |  |
| GSM972166 | 84.43779 | 0.438181 |  |  |  |
| GSM972142 | 88.00175 | 0.438407 |  |  |  |
| GSM972220 | 129.7197 | 0.438987 |  |  |  |
| GSM971988 | 122.9751 | 0.439285 |  |  |  |
| GSM972158 | 139.6225 | 0.43975 |  |  |  |
| GSM972236 | 112.8564 | 0.441304 |  |  |  |
| GSM972037 | 93.27268 | 0.442079 |  |  |  |
| GSM972143 | 77.2786 | 0.4426 |  |  |  |
| GSM972261 | 211.8831 | 0.443192 |  |  |  |
| GSM972410 | 126.8971 | 0.443962 |  |  |  |
| GSM972167 | 69.11569 | 0.445505 |  |  |  |
| GSM972521 | 144.7235 | 0.446344 |  |  |  |
| GSM972152 | 68.34429 | 0.447411 |  |  |  |
| GSM972012 | 83.95683 | 0.448509 |  |  |  |
| GSM972237 | 109.8741 | 0.450657 |  |  |  |
| GSM972194 | 81.85841 | 0.454716 |  |  |  |
| GSM972050 | 85.9359 | 0.456827 |  |  |  |
| GSM972106 | 133.7546 | 0.456983 |  |  |  |
| GSM972201 | 112.7693 | 0.460774 |  |  |  |
| GSM972445 | 329.5678 | 0.461493 |  |  |  |
| GSM971980 | 122.4852 | 0.463625 |  |  |  |
| GSM972110 | 128.197 | 0.463948 |  |  |  |
| GSM972385 | 160.5125 | 0.465619 |  |  |  |
| GSM972141 | 73.9514 | 0.467068 |  |  |  |
| GSM972406 | 63.28206 | 0.46844 |  |  |  |
| GSM972053 | 144.1316 | 0.469195 |  |  |  |
| GSM972432 | 105.2776 | 0.469372 |  |  |  |
| GSM972235 | 99.45803 | 0.469895 |  |  |  |
| GSM972051 | 124.0565 | 0.470914 |  |  |  |
| GSM972155 | 63.31195 | 0.471393 |  |  |  |
| GSM972414 | 72.79041 | 0.472611 |  |  |  |
| GSM972021 | 807.5414 | 0.47372 |  |  |  |
| GSM972266 | 195.7891 | 0.474373 |  |  |  |
| GSM972307 | 90.06494 | 0.475102 |  |  |  |
| GSM972114 | 84.98899 | 0.476202 |  |  |  |
| GSM972234 | 96.70832 | 0.476568 |  |  |  |
| GSM972359 | 96.68816 | 0.481536 |  |  |  |
| GSM972252 | 92.68329 | 0.482207 |  |  |  |
| GSM972435 | 77.58707 | 0.483542 |  |  |  |
| GSM971976 | 72.53191 | 0.486787 |  |  |  |
| GSM972286 | 159.8511 | 0.487423 |  |  |  |
| GSM972243 | 88.14616 | 0.490926 |  |  |  |
| GSM971997 | 143.8207 | 0.491381 |  |  |  |
| GSM972137 | 87.54824 | 0.491593 |  |  |  |
| GSM972268 | 99.00146 | 0.493809 |  |  |  |
| GSM972475 | 80.99756 | 0.49464 |  |  |  |
| GSM972383 | 104.7642 | 0.494839 |  |  |  |
| GSM972439 | 115.0701 | 0.495077 |  |  |  |
| GSM972508 | 113.3818 | 0.495678 |  |  |  |
| GSM972168 | 83.92964 | 0.496669 |  |  |  |
| GSM972485 | 132.8266 | 0.496764 |  |  |  |
| GSM972442 | 139.6134 | 0.496902 |  |  |  |
| GSM972520 | 72.86737 | 0.497916 |  |  |  |
| GSM972225 | 94.26034 | 0.497997 |  |  |  |
| GSM972007 | 160.8628 | 0.499316 |  |  |  |
| GSM972089 | 128.077 | 0.5013 |  |  |  |
| GSM972010 | 72.48388 | 0.501413 |  |  |  |
| GSM972032 | 139.5666 | 0.501838 |  |  |  |
| GSM972117 | 129.6666 | 0.501967 |  |  |  |
| GSM971979 | 116.1444 | 0.502058 |  |  |  |
| GSM972447 | 86.3206 | 0.502536 |  |  |  |
| GSM972154 | 99.8352 | 0.503226 |  |  |  |
| GSM972222 | 86.65729 | 0.50363 |  |  |  |
| GSM972064 | 74.42583 | 0.504608 |  |  |  |
| GSM972046 | 87.12633 | 0.507365 |  |  |  |
| GSM971985 | 96.78056 | 0.508685 |  |  |  |
| GSM972513 | 134.7967 | 0.510471 |  |  |  |
| GSM972440 | 135.1175 | 0.510595 |  |  |  |
| GSM972134 | 99.04744 | 0.512377 |  |  |  |
| GSM972233 | 72.11009 | 0.51344 |  |  |  |
| GSM972147 | 174.3774 | 0.515039 |  |  |  |
| GSM971974 | 138.4314 | 0.518121 |  |  |  |
| GSM972377 | 101.8461 | 0.51819 |  |  |  |
| GSM972198 | 80.44707 | 0.518777 |  |  |  |
| GSM972380 | 127.5466 | 0.519747 |  |  |  |
| GSM972229 | 123.9442 | 0.521234 |  |  |  |
| GSM972298 | 158.2299 | 0.521441 |  |  |  |
| GSM972119 | 87.20637 | 0.525597 |  |  |  |
| GSM972497 | 87.47507 | 0.526532 |  |  |  |
| GSM971961 | 92.94137 | 0.528513 |  |  |  |
| GSM972285 | 78.02491 | 0.529337 |  |  |  |
| GSM972045 | 78.53858 | 0.530894 |  |  |  |
| GSM971992 | 93.37891 | 0.532423 |  |  |  |
| GSM972231 | 83.72298 | 0.535091 |  |  |  |
| GSM972433 | 79.40541 | 0.535231 |  |  |  |
| GSM972041 | 101.385 | 0.542253 |  |  |  |
| GSM972327 | 100.7494 | 0.543258 |  |  |  |
| GSM972112 | 92.25797 | 0.543955 |  |  |  |
| GSM972206 | 86.56749 | 0.545025 |  |  |  |
| GSM972165 | 70.75034 | 0.546065 |  |  |  |
| GSM972054 | 110.6385 | 0.548558 |  |  |  |
| GSM972463 | 58.25803 | 0.549472 |  |  |  |
| GSM972388 | 123.6072 | 0.551358 |  |  |  |
| GSM972035 | 95.77162 | 0.551669 |  |  |  |
| GSM972405 | 68.69021 | 0.552378 |  |  |  |
| GSM971964 | 82.50533 | 0.553381 |  |  |  |
| GSM972048 | 86.81453 | 0.553621 |  |  |  |
| GSM972477 | 86.34993 | 0.553915 |  |  |  |
| GSM972354 | 94.9372 | 0.55667 |  |  |  |
| GSM972001 | 90.70552 | 0.559344 |  |  |  |
| GSM972091 | 98.69063 | 0.560792 |  |  |  |
| GSM972200 | 78.97292 | 0.562595 |  |  |  |
| GSM972365 | 122.4708 | 0.563327 |  |  |  |
| GSM972094 | 63.67646 | 0.564289 |  |  |  |
| GSM972342 | 77.11502 | 0.564509 |  |  |  |
| GSM972494 | 125.4961 | 0.564734 |  |  |  |
| GSM972164 | 91.24927 | 0.565108 |  |  |  |
| GSM971977 | 91.64646 | 0.5656 |  |  |  |
| GSM972509 | 111.7317 | 0.565686 |  |  |  |
| GSM972005 | 102.2938 | 0.56765 |  |  |  |
| GSM972408 | 73.98402 | 0.568619 |  |  |  |
| GSM972355 | 81.40284 | 0.56957 |  |  |  |
| GSM972090 | 84.34522 | 0.570371 |  |  |  |
| GSM972507 | 183.7413 | 0.570603 |  |  |  |
| GSM972264 | 180.4806 | 0.571008 |  |  |  |
| GSM972213 | 78.91773 | 0.571603 |  |  |  |
| GSM972402 | 58.99685 | 0.572917 |  |  |  |
| GSM972467 | 141.2852 | 0.573042 |  |  |  |
| GSM971990 | 86.02408 | 0.574912 |  |  |  |
| GSM972462 | 93.48283 | 0.574942 |  |  |  |
| GSM972374 | 145.1292 | 0.576303 |  |  |  |
| GSM972189 | 152.724 | 0.577343 |  |  |  |
| GSM972289 | 68.92847 | 0.577806 |  |  |  |
| GSM972078 | 83.04317 | 0.578809 |  |  |  |
| GSM972496 | 115.6719 | 0.579981 |  |  |  |
| GSM972369 | 125.1105 | 0.581522 |  |  |  |
| GSM972159 | 95.86723 | 0.582465 |  |  |  |
| GSM972434 | 81.63321 | 0.585304 |  |  |  |
| GSM972203 | 98.36521 | 0.586073 |  |  |  |
| GSM972092 | 129.3016 | 0.587203 |  |  |  |
| GSM972517 | 105.4167 | 0.587237 |  |  |  |
| GSM972196 | 102.202 | 0.587279 |  |  |  |
| GSM972004 | 88.16192 | 0.588311 |  |  |  |
| GSM971969 | 146.0672 | 0.589055 |  |  |  |
| GSM972074 | 76.53739 | 0.592399 |  |  |  |
| GSM972009 | 85.11428 | 0.592637 |  |  |  |
| GSM972242 | 85.98005 | 0.593036 |  |  |  |
| GSM972344 | 64.10704 | 0.595039 |  |  |  |
| GSM972483 | 71.12301 | 0.596001 |  |  |  |
| GSM972221 | 145.0744 | 0.597615 |  |  |  |
| GSM972278 | 148.9876 | 0.597814 |  |  |  |
| GSM972455 | 84.24222 | 0.599414 |  |  |  |
| GSM972400 | 111.8989 | 0.59986 |  |  |  |
| GSM972389 | 129.4407 | 0.600651 |  |  |  |
| GSM972132 | 68.84768 | 0.602065 |  |  |  |
| GSM972438 | 87.48571 | 0.602229 |  |  |  |
| GSM972401 | 150.0841 | 0.604936 |  |  |  |
| GSM972326 | 81.42604 | 0.605354 |  |  |  |
| GSM972008 | 136.5582 | 0.60625 |  |  |  |
| GSM972202 | 78.49215 | 0.607125 |  |  |  |
| GSM972356 | 92.44032 | 0.607379 |  |  |  |
| GSM972066 | 78.97224 | 0.608423 |  |  |  |
| GSM972070 | 66.6597 | 0.608528 |  |  |  |
| GSM972464 | 86.62839 | 0.609058 |  |  |  |
| GSM972218 | 94.87358 | 0.610451 |  |  |  |
| GSM972197 | 115.7344 | 0.610885 |  |  |  |
| GSM972514 | 110.3001 | 0.611223 |  |  |  |
| GSM972361 | 90.46777 | 0.611554 |  |  |  |
| GSM972014 | 114.8477 | 0.612443 |  |  |  |
| GSM972512 | 118.1785 | 0.613984 |  |  |  |
| GSM972306 | 80.39364 | 0.61536 |  |  |  |
| GSM972431 | 86.71878 | 0.615394 |  |  |  |
| GSM972386 | 83.20838 | 0.619467 |  |  |  |
| GSM972469 | 111.6706 | 0.61968 |  |  |  |
| GSM972491 | 63.71646 | 0.621379 |  |  |  |
| GSM971975 | 87.71496 | 0.624052 |  |  |  |
| GSM972043 | 72.13769 | 0.625081 |  |  |  |
| GSM972437 | 100.619 | 0.625298 |  |  |  |
| GSM972124 | 98.66063 | 0.625991 |  |  |  |
| GSM971993 | 111.6206 | 0.629566 |  |  |  |
| GSM972384 | 91.59335 | 0.631054 |  |  |  |
| GSM972088 | 79.94388 | 0.635342 |  |  |  |
| GSM972208 | 112.7545 | 0.637364 |  |  |  |
| GSM972411 | 84.55844 | 0.637586 |  |  |  |
| GSM971987 | 88.61697 | 0.641025 |  |  |  |
| GSM972448 | 85.26207 | 0.64204 |  |  |  |
| GSM971962 | 113.5415 | 0.64373 |  |  |  |
| GSM972486 | 74.87973 | 0.646398 |  |  |  |
| GSM972108 | 68.40129 | 0.64687 |  |  |  |
| GSM972315 | 81.9323 | 0.648476 |  |  |  |
| GSM972329 | 76.92631 | 0.649883 |  |  |  |
| GSM972320 | 63.50474 | 0.651442 |  |  |  |
| GSM972350 | 76.51281 | 0.651482 |  |  |  |
| GSM972453 | 75.78434 | 0.652067 |  |  |  |
| GSM972511 | 84.16156 | 0.656386 |  |  |  |
| GSM972322 | 86.72299 | 0.656802 |  |  |  |
| GSM972403 | 76.47847 | 0.658006 |  |  |  |
| GSM972312 | 82.28537 | 0.659266 |  |  |  |
| GSM972084 | 76.44858 | 0.660294 |  |  |  |
| GSM972013 | 87.00064 | 0.660443 |  |  |  |
| GSM972103 | 131.3044 | 0.660981 |  |  |  |
| GSM972352 | 78.72266 | 0.661844 |  |  |  |
| GSM972068 | 81.92878 | 0.662409 |  |  |  |
| GSM972349 | 88.79549 | 0.664116 |  |  |  |
| GSM972456 | 85.16319 | 0.665032 |  |  |  |
| GSM972209 | 110.5579 | 0.666123 |  |  |  |
| GSM972120 | 139.2429 | 0.666544 |  |  |  |
| GSM972085 | 76.54582 | 0.668371 |  |  |  |
| GSM972238 | 108.122 | 0.669746 |  |  |  |
| GSM971965 | 95.78526 | 0.671817 |  |  |  |
| GSM972067 | 93.92319 | 0.672614 |  |  |  |
| GSM972358 | 104.5755 | 0.67373 |  |  |  |
| GSM971984 | 89.47089 | 0.673823 |  |  |  |
| GSM972109 | 308.3918 | 0.677365 |  |  |  |
| GSM972095 | 92.37337 | 0.677812 |  |  |  |
| GSM971972 | 77.56571 | 0.678785 |  |  |  |
| GSM972281 | 92.42507 | 0.679198 |  |  |  |
| GSM972325 | 70.76285 | 0.680056 |  |  |  |
| GSM972302 | 53.10388 | 0.680086 |  |  |  |
| GSM971981 | 114.4272 | 0.680818 |  |  |  |
| GSM972128 | 75.29088 | 0.681188 |  |  |  |
| GSM972407 | 81.50235 | 0.68165 |  |  |  |
| GSM972303 | 63.74894 | 0.682229 |  |  |  |
| GSM972020 | 80.99756 | 0.68333 |  |  |  |
| GSM972480 | 75.88578 | 0.683706 |  |  |  |
| GSM972360 | 113.6348 | 0.683893 |  |  |  |
| GSM972343 | 83.33239 | 0.684413 |  |  |  |
| GSM972082 | 65.5619 | 0.684782 |  |  |  |
| GSM972368 | 116.2904 | 0.684806 |  |  |  |
| GSM972042 | 96.10831 | 0.68543 |  |  |  |
| GSM972081 | 91.93316 | 0.687882 |  |  |  |
| GSM972476 | 80.80458 | 0.688977 |  |  |  |
| GSM972060 | 69.42484 | 0.689631 |  |  |  |
| GSM972470 | 100.3103 | 0.693692 |  |  |  |
| GSM972056 | 93.16647 | 0.694588 |  |  |  |
| GSM972083 | 67.24708 | 0.695085 |  |  |  |
| GSM972038 | 108.4507 | 0.699951 |  |  |  |
| GSM971958 | 90.15715 | 0.700286 |  |  |  |
| GSM972324 | 75.03822 | 0.701301 |  |  |  |
| GSM972314 | 95.82329 | 0.701872 |  |  |  |
| GSM972047 | 81.72556 | 0.702377 |  |  |  |
| GSM972212 | 74.32917 | 0.702871 |  |  |  |
| GSM972459 | 83.5549 | 0.703041 |  |  |  |
| GSM971963 | 194.8616 | 0.703512 |  |  |  |
| GSM972214 | 92.52328 | 0.704731 |  |  |  |
| GSM972055 | 66.58024 | 0.706535 |  |  |  |
| GSM972479 | 119.1689 | 0.70843 |  |  |  |
| GSM972458 | 116.6721 | 0.708844 |  |  |  |
| GSM972487 | 72.0863 | 0.709146 |  |  |  |
| GSM971968 | 96.32822 | 0.709554 |  |  |  |
| GSM972457 | 52.55395 | 0.709573 |  |  |  |
| GSM972052 | 124.2732 | 0.711836 |  |  |  |
| GSM972040 | 67.26764 | 0.714432 |  |  |  |
| GSM972215 | 131.8734 | 0.714641 |  |  |  |
| GSM972353 | 117.5221 | 0.71682 |  |  |  |
| GSM972334 | 112.1205 | 0.716932 |  |  |  |
| GSM972069 | 51.10745 | 0.716951 |  |  |  |
| GSM972363 | 87.72369 | 0.717257 |  |  |  |
| GSM972049 | 102.3263 | 0.717376 |  |  |  |
| GSM972058 | 78.03118 | 0.719097 |  |  |  |
| GSM972465 | 75.56983 | 0.71936 |  |  |  |
| GSM972482 | 74.54452 | 0.719391 |  |  |  |
| GSM972489 | 87.27006 | 0.724296 |  |  |  |
| GSM972409 | 83.24188 | 0.726107 |  |  |  |
| GSM972338 | 81.32932 | 0.729975 |  |  |  |
| GSM972345 | 73.96512 | 0.731306 |  |  |  |
| GSM972073 | 71.21492 | 0.732287 |  |  |  |
| GSM971999 | 107.7823 | 0.732768 |  |  |  |
| GSM972376 | 88.63806 | 0.733713 |  |  |  |
| GSM972309 | 65.12856 | 0.733784 |  |  |  |
| GSM971966 | 84.35397 | 0.735557 |  |  |  |
| GSM972451 | 67.57762 | 0.736938 |  |  |  |
| GSM972335 | 75.93335 | 0.737434 |  |  |  |
| GSM972313 | 108.3091 | 0.739433 |  |  |  |
| GSM972319 | 124.8886 | 0.740307 |  |  |  |
| GSM972515 | 74.49417 | 0.741233 |  |  |  |
| GSM972063 | 77.65534 | 0.743441 |  |  |  |
| GSM972372 | 103.2041 | 0.744382 |  |  |  |
| GSM972340 | 84.03981 | 0.744446 |  |  |  |
| GSM972323 | 79.28302 | 0.745664 |  |  |  |
| GSM971982 | 142.7137 | 0.745998 |  |  |  |
| GSM972207 | 92.46449 | 0.746235 |  |  |  |
| GSM972076 | 67.11217 | 0.746434 |  |  |  |
| GSM971973 | 87.52302 | 0.747569 |  |  |  |
| GSM972308 | 98.10523 | 0.748164 |  |  |  |
| GSM972466 | 82.4896 | 0.749209 |  |  |  |
| GSM972460 | 94.32722 | 0.749533 |  |  |  |
| GSM972332 | 69.22337 | 0.74974 |  |  |  |
| GSM972493 | 83.63979 | 0.749862 |  |  |  |
| GSM972472 | 72.29637 | 0.750073 |  |  |  |
| GSM971957 | 113.2121 | 0.75091 |  |  |  |
| GSM971978 | 96.20655 | 0.755531 |  |  |  |
| GSM972104 | 119.3434 | 0.757395 |  |  |  |
| GSM972351 | 68.84436 | 0.757415 |  |  |  |
| GSM972454 | 88.67981 | 0.759041 |  |  |  |
| GSM972016 | 83.01213 | 0.760263 |  |  |  |
| GSM972347 | 78.21707 | 0.766556 |  |  |  |
| GSM971970 | 99.19038 | 0.766819 |  |  |  |
| GSM972087 | 82.79263 | 0.767422 |  |  |  |
| GSM972330 | 79.48 | 0.770141 |  |  |  |
| GSM971994 | 66.99617 | 0.777565 |  |  |  |
| GSM972387 | 74.36622 | 0.778042 |  |  |  |
| GSM972461 | 64.75472 | 0.778086 |  |  |  |
| GSM972318 | 67.0287 | 0.778667 |  |  |  |
| GSM972062 | 62.31332 | 0.779766 |  |  |  |
| GSM972337 | 83.79327 | 0.781151 |  |  |  |
| GSM971971 | 132.7321 | 0.783706 |  |  |  |
| GSM972129 | 145.6488 | 0.784734 |  |  |  |
| GSM972339 | 70.64398 | 0.784792 |  |  |  |
| GSM972044 | 64.32329 | 0.786025 |  |  |  |
| GSM972488 | 70.70445 | 0.787235 |  |  |  |
| GSM972364 | 79.09067 | 0.787687 |  |  |  |
| GSM972498 | 76.56505 | 0.788801 |  |  |  |
| GSM972346 | 87.22438 | 0.792823 |  |  |  |
| GSM972478 | 78.2257 | 0.794923 |  |  |  |
| GSM972362 | 134.0973 | 0.798276 |  |  |  |
| GSM971959 | 80.47268 | 0.801484 |  |  |  |
| GSM971983 | 95.37133 | 0.801963 |  |  |  |
| GSM972080 | 73.29726 | 0.805637 |  |  |  |
| GSM972499 | 92.74329 | 0.805804 |  |  |  |
| GSM971967 | 108.8867 | 0.807595 |  |  |  |
| GSM972316 | 72.6391 | 0.80849 |  |  |  |
| GSM972075 | 77.04982 | 0.811224 |  |  |  |
| GSM971960 | 72.88527 | 0.811513 |  |  |  |
| GSM972481 | 76.10591 | 0.814453 |  |  |  |
| GSM972310 | 87.14424 | 0.815462 |  |  |  |
| GSM972071 | 63.13663 | 0.818423 |  |  |  |
| GSM972336 | 75.35932 | 0.822118 |  |  |  |
| GSM972449 | 111.1434 | 0.827483 |  |  |  |
| GSM972474 | 104.9923 | 0.835754 |  |  |  |
| GSM972468 | 72.57913 | 0.840085 |  |  |  |
| GSM972328 | 71.66582 | 0.844611 |  |  |  |
| GSM972072 | 103.1656 | 0.845468 |  |  |  |
| GSM972450 | 91.48298 | 0.846746 |  |  |  |
| GSM972473 | 70.85839 | 0.847637 |  |  |  |
| GSM972065 | 66.50458 | 0.84829 |  |  |  |
| GSM972490 | 51.85952 | 0.85319 |  |  |  |
| GSM972484 | 62.71468 | 0.854262 |  |  |  |
| GSM972471 | 77.73237 | 0.85451 |  |  |  |
| GSM972015 | 109.4885 | 0.860658 |  |  |  |
| GSM972086 | 73.15761 | 0.861293 |  |  |  |
| GSM972404 | 74.61857 | 0.864897 |  |  |  |
| GSM972492 | 65.85418 | 0.866759 |  |  |  |
| GSM972333 | 76.50247 | 0.874794 |  |  |  |
| GSM972452 | 111.6707 | 0.876723 |  |  |  |
| GSM972002 | 78.08043 | 0.881502 |  |  |  |
| GSM972348 | 74.35418 | 0.886716 |  |  |  |
| GSM972341 | 93.57693 | 0.890751 |  |  |  |
| GSM972331 | 88.60264 | 0.891575 |  |  |  |
| GSM972321 | 58.51309 | 0.90678 |  |  |  |
| GSM972317 | 80.66238 | 0.907762 |  |  |  |
